# Dose-Ranging Effects of the Intracerebral Administration of Atsttrin in Experimental Model of Parkinson’s Disease Induced by 1-Methyl-4-phenyl-1,2,3,6-tetrahydropyridine (MPTP) in Mice

**DOI:** 10.1007/s12035-024-04161-0

**Published:** 2024-04-20

**Authors:** Łukasz A. Poniatowski, Ilona Joniec-Maciejak, Adriana Wawer, Anna Sznejder-Pachołek, Ewa Machaj, Katarzyna Ziętal, Dagmara Mirowska-Guzel

**Affiliations:** 1 Department of Experimental and Clinical Pharmacology, Centre for Preclinical Research and Technology (CePT), Medical University of Warsaw, Banacha 1B, 02-097 Warsaw, Poland; 2 Department of Neurosurgery, Dietrich-Bonhoeffer-Klinikum, Salvador-Allende-Straße 30, 17036 Neubrandenburg, Germany

**Keywords:** Atsttrin, Progranulin, Parkinson’s disease, Neurodegeneration, MPTP, Inflammatory reaction

## Abstract

Parkinson’s disease is one of the most common neurodegenerative disorders characterized by a multitude of motor and non-motor clinical symptoms resulting from the progressive and long-lasting abnormal loss of nigrostriatal dopaminergic neurons. Currently, the available treatments for patients with Parkinson’s disease are limited and exert only symptomatic effects, without adequate signs of delaying or stopping the progression of the disease. Atsttrin constitutes the bioengineered protein which ultrastructure is based on the polypeptide chain frame of the progranulin (PGRN), which exerts anti-inflammatory effects through the inhibition of TNFα. The conducted preclinical studies suggest that the therapeutic implementation of Atsttrin may be potentially effective in the treatment of neurodegenerative diseases that are associated with the occurrence of neuroinflammatory processes. The aim of the proposed study was to investigate the effect of direct bilateral intracerebral administration of Atsttrin using stereotactic methods in the preclinical C57BL/6 mouse model of Parkinson’s disease inducted by 1-methyl-4-phenyl-1,2,3,6-tetrahydropyridine (MPTP) intoxication. The analysis of the dose dependency effects of the increasing doses of Atsttrin has covered a number of parameters and markers regarding neurodegenerative processes and inflammatory responses including IL-1α, TNFα, IL-6, TH, and TG2 mRNA expressions. Accordingly, the evaluation of the changes in the neurochemical profile included DA, DOPAC, 3-MT, HVA, NA, MHPG, 5-HT, and 5-HIAA concentration levels. The intracerebral administration of Atsttrin into the striatum effectively attenuated the neuroinflammatory reaction in evaluated neuroanatomical structures. Furthermore, the partial restoration of monoamine content and its metabolic turnover were observed. In this case, taking into account the previously described pharmacokinetic profile and extrapolated bioavailability as well as the stability characteristics of Atsttrin, an attempt was made to describe as precisely as possible the quantitative and qualitative effects of increasing doses of the compound within the brain tissue microenvironment in the presented preclinical model of the disease. Collectively, this findings demonstrated that the intracerebral administration of Atsttrin may represent a potential novel therapeutic method for the treatment of Parkinson’s disease.

**Supplementary Information:**

The online version contains supplementary material available at 10.1007/s12035-024-04161-0.

## Introduction

Parkinson’s disease constitutes one of the most common neurodegenerative disorders of the central nervous system (CNS), which clinically involves motor function considered to be the core symptom which is associated with the subsequent gradually appearing non-motor complications [1, 2]. The characteristic clinical symptoms constitute slowness of movement (bradykinesia), rigidity, tremor, and gait and posture abnormalities [3]. Non-motor symptoms of Parkinson’s disease are associated with cognitive impairment, sleep disturbances, autonomic dysfunctions, and neuropsychiatric abnormalities. The pathomechanism of Parkinson’s disease is characterized by the primary progressive and long-lasting abnormal loss of ventrolateral and caudal portions of nigrostriatal dopaminergic neurons situated in the pars compacta of substantia nigra (SN) within the midbrain [4]. The loss of nerve terminals is accompanied by the gradual depletion of dopamine (DA) and its metabolite concentration in the basal ganglia, including the striatum (ST), which leads in the direct way to disturbances in the homeostasis of the neurotransmitter system and the occurrence of various neurological symptoms [5]. According to the studies and accumulating evidence from the last decade, it becomes evident that the immune system and neuroinflammatory response play a significant role in the pathogenesis of Parkinson’s disease [6]. It was observed that the ongoing neurodegenerative process in the nigrostriatal pathway is connected with the excessive local and systemic immunoactivations involving multiple inflammatory cells and their related mediators [7].

Recently, investigations around the group of anti-inflammatory cytokines and their based derivatives have pointed a promising and innovating direction in the treatment of neuroinflammatory-related conditions covering Parkinson’s disease, potentially supporting either the protective or regenerative properties of the neural tissue. Recent advances in this field have been made with the investigation of novel multifunctional growth factor progranulin (PGRN), which has attracted a significant attention throughout the neuroscience community on account of its potent and specific neurotrophic, anti-inflammatory, and immunomodulatory features [8]. In this case, the two cutting-edge studies published in the year 2006 showed that haploinsufficiency and null mutations of the PGRN gene were recognized as a determinant of diverse familiar forms of frontotemporal lobar degeneration (FTLD), sharing the neuropathology of neural intracellular inclusions, proteinopathy, and the trigger landmark research aimed at explaining PGRN function in the CNS [9, 10]. Regarding the pathologies within the CNS, dysregulation of PGRN functioning associated with the homozygous and heterozygous nonsense, frameshift, and splice-site mutations were to date potentially linked to the development of Alzheimer’s disease, amyotrophic lateral sclerosis, motor neuron disease, neuronal ceroid lipofuscinosis, Creutzfeldt-Jakob disease, epilepsy, bipolar disorder, and schizophrenia [11, 12]. In a larger context, it seems that PGRN is functioning as a physiological and potentially critical controller of homeostasis and neuronal functions, a neurotrophic factor which regulates neurite outgrowth and maintains its survival [13, 14]. Various published studies describing the newly discovered phenomena in regard to the competitive influence of endogenous PGRN and its related smaller polypeptides on receptors associated with tumor necrosis factor alpha (TNFα)/tumor necrosis factor receptor 1/2 (TNFR1/2) interactions as well as other multiple interactions and interplay with, e.g., cytokines, chemokines, proteinases growth factors, and cellular receptors indicate the main crucial mechanism of PGRN participation in pathophysiological actions that precisely influence and orchestrate neuroinflammatory phenomena [15]. Therefore, the accumulating knowledge about PGRN and its associated multiple receptors and signaling pathways implies that behind distinct pathologies PGRN could constitute a critical molecule essential in the biological functioning of all cells within the CNS [16].

Atsttrin constitutes the new bioengineered protein which ultrastructure is based on the polypeptide chain frame of three PGRN domains in order F-A-C (1/2F-P3-P4-1/2A-P5-1/2C) which are responsible and required for direct binding and effect on TNFR1 and TNFR2 receptors [17]. According to the performed kinetic studies using surface plasmon resonance (SPR) analysis, it was observed that PGRN, and potentially Atsttrin, could bind to TNFR1 with affinity comparable to TNFα and bind to TNFR2 with ~ 600-fold higher affinity than TNFα itself in a dose-dependent manner [17, 18]. Importantly, Atsttrin binds to TNFR1 with ~ 18-fold lower affinity than TNFα and binds to TNFR2 with ~ tenfold higher than TNFα itself. In this case, the anti-inflammatory effect of Atsttrin in the pharmacodynamic sense should potentially exceed PGRN and other well-known anti-TNFα factors. Moreover, pharmacodynamic properties maintaining kinetic binding affinity to TNFR1/2 potentially avoid disadvantages associated with the exhibition of cytokine and growth factor–like and potential oncogenic properties contrary to other TNFα-blockers such as infliximab and adalimumab, potentially exerting a tumor-suppressing activity as well [17–20]. In the collagen-induced arthritis (CIA) model of arthritis in the C57BL/6 mice, the significant and practically complete regression of joint destruction and associated inflammation was observed compared to control animals which did not receive intraperitoneal Atsttrin (0.1–5.0 mg/kg body weight) injection [17]. Similar observations have been described for the mouse oxazolone (OXA)-induced dermatitis model, which also confirmed the anti-inflammatory effect of Atsttrin (2.5 mg/kg body weight) and discussed the use of this protein as a potential drug in the prevention and treatment of inflammatory skin diseases [21]. In addition to the protective effect of Atsttrin on joint tissues and the skin, a positive effect was also observed in ex vivo studies on mouse and human tissues derived from intervertebral discs incubated with Atsttrin (1 µg/mL for 7 days), proposing the protein as a potential drug in the prevention and treatment of degenerative disc disease [22].

Conducted preclinical studies suggest in this case that the implementation of Atsttrin for therapy may be potentially effective in the treatment of diseases of the CNS that are associated with the occurrence of neuroinflammatory process. Recently, it was observed that C57BL/6 mice subjected to intracerebroventricular injection of 1 μL (10 μg/μL) lipopolysaccharide (LPS) and subsequent intraperitoneal administration of Atsttrin (2.5 mg/kg every 3 days over a period of 7 days) have presented reduced LPS-induced mRNA increase of TNFα, interleukin 1 beta (IL-1β), matrix metalloproteinase 3 (MMP-3), inducible nitric oxide synthase (iNOS), and cyclooxygenase 2 (COX-2) [23]. Additionally, administration of Atsttrin in this model significantly reduced the levels of phospho-nuclear factor kappa B (NF-κB) in the brain of LPS-treated PGRN knockout mice and cultured astrocyte cells. Collectively, the pharmacodynamic profile of Atsttrin could potentially replicate the autologous PGRN mechanisms as neurotrophic factor which regulate neuronal functions and neurite outgrowth, maintaining its survival. In this case, Atsttrin could potentially surpass another well-known neurotropic factor, presenting both unique both growth factor–like and anti-inflammatory properties.

In line with these findings, the concept pertaining to the local attenuation of neuroinflammatory response via direct intracerebral administration of PGRN-based agents using stereotactic methods poses an interesting potential therapeutic strategy which could potentially contribute to the slowdown of Parkinson’s disease and may lead to the improvement in the clinical status of patients suffering from the disease. The preclinical protocols assuming the use of 1-methyl-4-phenyl-1,2,3,6-tetrahydropyridine (MPTP) constitute the most common neurotoxin-based models, used mainly in non‐human primates and in mice that are applied in Parkinson’s disease research [24]. Neurochemical and histopathological investigations have to date demonstrated that MPTP‐induced parkinsonism reproduces the pathophysiology of human disease and has been valuable for the evaluation of the effects of various drug therapies and efficacy of new surgical techniques [25]. The aim of the proposed study is to investigate the effect of direct bilateral intracerebral infusion of Atsttrin using stereotactic methods in the preclinical C57BL/6 mouse model of Parkinson’s disease inducted by MPTP intoxication. To date, the potential evaluation and dose optimization of Atsttrin in this experimental model were not previously evaluated in neurosciences field. In this case, we will introduce and validate the method of intracerebral stereotactic Atsttrin delivery relative to MPTP intoxication and estimate the most effective and characterized by a high level of safety dose of Atsttrin based on the response curve. The dose-dependent evaluation has covered a number of parameters and markers regarding neurodegenerative processes and development of inflammatory responses including TNFα, interleukin 1 alpha (IL-1α), interleukin 6 (IL-6), tyrosine hydroxylase (TH), and transglutaminase 2 (TG2) mRNA expressions. In this case, we have also evaluated the changes in neurochemical profile including the concentration of monoamines such as DA and its metabolites such as 3,4-dihydroxyphenylacetic acid (DOPAC), 3-methoxytyramine (3-MT), homovanillic acid (HVA), norepinephrine (NA), 3-methoxy-4-hydroxyphenylglycol (MHPG), serotonin (5-HT), and 5-hydroxyindoleacetic acid (5-HIAA).

## Materials and Methods

### Animals

Inbred male C57BL/6 mice at the age of 10–12 months (body weight of 30 ± 5 g) were used in this study. All animals were housed in plastic cages (1290; Tecniplast, Varese, Italy) in groups of 3–5/cage in standard laboratory conditions at a controlled temperature (22 ± 5 °C) and 60 ± 5% humidity (15 air changes/h) with a 12-h light/dark cycle (7:00 a.m./7:00 p.m.) and constant lighting intensity. The animals were provided access to food with dietary standards of the Nutrient Requirement of Laboratory Animals (4th Revised Edition, 1995; National Academies Press, Washington, DC, USA) and water ad libitum [26]. Before performing invasive experimental procedures, animals were separated to appropriate cages and groups based on the study protocol and subjected to 2-day habituation to obtain hormonal and neurochemical stabilities [27]. All efforts were made to reduce the number of animals used and to minimize animals’ discomfort and suffering through a practical implementation of the 3R principle (replacement, reduction, and refinement) [28, 29].

### Experimental Design

**Fig. 1:**
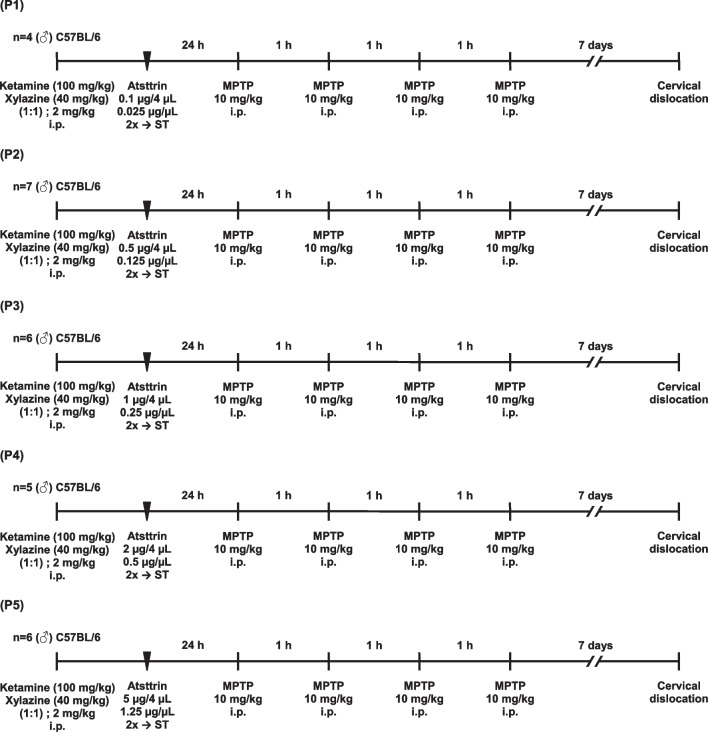
Schematic timelines showing the experiment design and procedures performed on study groups which were administrated intracerebrally with predefined increasing dose of Atsttrin and subjected to MPTP intoxication (P1–P5). 2 ×  → ST, bilateral intracerebral administration of Atsttrin into the striatum using stereotactic methods; MPTP, 1-methyl-4-phenyl-1,2,3,6-tetrahydropyridine; i.p., intraperitoneal injection

**Fig. 2:**
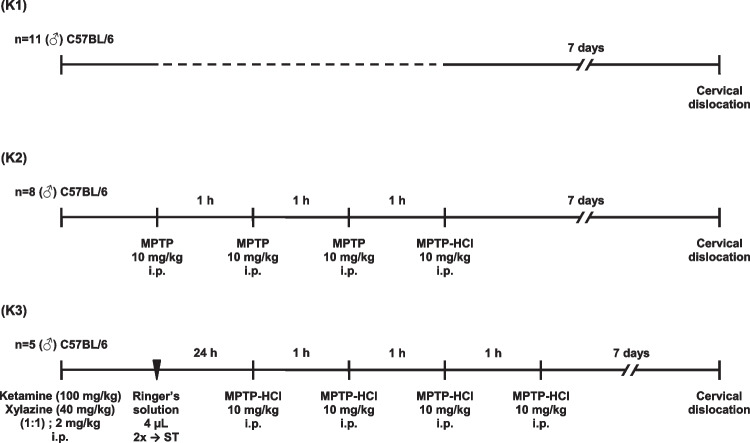
Schematic timelines showing the intervention design and procedures performed on control groups (K1–K3). 2 ×  → ST, bilateral intracerebral administration of Ringer’s solution into the striatum using stereotactic methods; MPTP, 1-methyl-4-phenyl-1,2,3,6-tetrahydropyridine; i.p., intraperitoneal injection

According to the systematic literature review, to date, no attempts had been made regarding the intracerebral administration of Atsttrin. This also implied the existing lack of knowledge enabling precise dose optimization and validation of Atsttrin administered potentially this route and in this experimental model. A predefined range value of five increasing Atsttrin doses of 0.1 μg (0.025 μg/μL), 0.5 μg (0.125 μg/μL), 1 μg (0.25 μg/μL), 2 μg (0.5 μg/μL), and 5 μg (1.25 μg/μL) was extrapolated and determined on the basis of literature data from previously conducted studies on the musculoskeletal system in mice [17]. In this case, the dose values and concentration of the Atsttrin were determined using the scaling method based on the animal body weight and taking into account the correction of existing anatomical limitations related to the intracerebral administration method using stereotaxic methods taking into account the size and location of neuroanatomical targets structures, proximity of critical areas, and possible avoidance of the ablative effect [30]. Taking into account the range of Atsttrin doses described in available studies and the average weight of C57BL/6 strain mice at the age of 10–12 months of 30 ± 5 g, it can be assumed that the potential therapeutic dose of the compound based on the data available in the literature amounts potentially to ~ 0.075 to 0.15 mg. Due to the preliminary nature of this study, the injection and was limited to one stereotaxic target covering ST, which includes the most prominent and largest neuroanatomical structure among the basal ganglia and subcortical structures, thus being a relatively accessible structure regarding stereotactic procedures, with a significant volume compared to other parts of the mouse brain [31, 32]. Regarding the neurophysiological functions, the initial choice of ST as a neuroanatomical target was dedicated by its role as the main center integrating and regulating signals within the nigrostriatal system which is mainly affected in the course of Parkinson’s disease [33]. According to the study protocol, the experimental procedures followed by subsequent molecular and biochemical analyses were performed on a total number of 52 animals (*n* = 52) covering male C57BL/6 mice, which amount was precisely estimated using a publicly available online calculator (http://www.biomath.info/power) [34]. In order to optimize and validate the dose of Atsttrin administered intracerebrally, as well as provide novel insights regarding evaluated murine model of Parkinson’s disease, corresponding study groups were defined (Fig. 1). The study group consisted of a total number of 28 animals (*n* = 28) subjected to MPTP intoxication which were consecutively allocated in each of the five subgroups (P1–P5) on the basis of the predefined increasing dose of Atsttrin which was administered intracerebrally using stereotactic methods (Table 1). In order to properly interpret the obtained results, as well as to provide basic reference and verification of the methods used throughout the experiments, corresponding control groups were defined (Fig. 2). The control group consisted of a total number of 24 animals (*n* = 24) which were appropriately divided into three subgroups (K1–K3), taking into account the type of performed experimental procedure and intervention requiring verification (Table 2).

**Table 1:** Characteristics and division of study groups according to the predefined increasing doses of Atsttrin which was administered intracerebrally using stereotactic methods

Group	Number of animals (*n*)	Code
Atsttrin 0.1 μg/4 μL (0.025 μg/μL) → ST + 4 × MPTP (7d)	4	P1
Atsttrin 0.5 μg/4 μL (0.125 μg/μL) → ST + 4 × MPTP (7d)	7	P2
Atsttrin 1 μg/4 μL (0.25 μg/μL) → ST + 4 × MPTP (7d)	6	P3
Atsttrin 2 μg/4 μL (0.5 μg/μL) → ST + 4 × MPTP (7d)	5	P4
Atsttrin 5 μg/4 μL (1.25 μg/μL) → ST + 4 × MPTP (7d)	6	P5

7d, an assumed 7-day time point at which animals were sacrificed in accordance with the study protocol; 4 × MPTP, four serial intraperitoneal injections of 1-methyl-4-phenyl-1,2,3,6-tetrahydropyridine; Atsttrin 0.1 μg/4 μL (0.025 μg/μL) → ST, bilateral intracerebral administration of Atsttrin at a dose of 0.1 μg/4 μL (0.025 μg/μL) into the striatum using stereotactic methods; Atsttrin 0.5 μg/4 μL (0.125 μg/μL) → ST, bilateral intracerebral administration of Atsttrin at a dose of 0.5 μg/4 μL (0.125 μg/μL) into the striatum using stereotactic methods; Atsttrin 1 μg/4 μL (0.25 μg/μL) → ST, bilateral intracerebral administration of Atsttrin at a dose of 1 μg/4 μL (0.25 μg/μL) into the striatum using stereotactic methods; Atsttrin 2 μg/4 μL (0.5 μg/μL) → ST, bilateral intracerebral administration of Atsttrin at a dose of 2 μg/4 μL (0.5 μg/μL) into the striatum using stereotactic methods; Atsttrin 5 μg/4 μL (1.25 μg/μL) → ST, bilateral intracerebral administration of Atsttrin at a dose of 5 μg/4 μL (1.25 μg/μL) into the striatum using stereotactic methods

**Table 2:** Characteristics and division of control groups according to the type of performed procedure and intervention requiring verification

Group	Number of animals (*n*)	Code
Control group not subjected to any procedure or intervention (7d)	11	K1
4 × MPTP (7d)	8	K2
Ringer’s solution (4 μL) → ST + 4 × MPTP (7d)	5	K3

7d, an assumed 7-day time point at which animals were sacrificed in accordance with the study protocol; 4 × MPTP, four serial intraperitoneal injections of 1-methyl-4-phenyl-1,2,3,6-tetrahydropyridine; Ringer’s solution (4 μL) → ST, bilateral intracerebral administration of Ringer’s solution (4 μL) into the striatum using stereotactic methods

### Stereotactic Injections

The animals were anesthetized with ketamine (Ketanest 50 mg/mL; Pfizer, New York, NY, USA) and xylazine (Xylapan 20 mg/mL; Vetoquinol AG, Bern, Switzerland) combination (1:1, v/v) in the dose of 2 mL/kg body weight (100 mg/kg ketamine and 40 mg/kg xylazine) via intraperitoneal administration using a 25G × 5/8″ (∅ 0.5 × 16 mm) needle with a 1-mL syringe (300,014; BD Plastipak, Madrid, Spain) under aseptic conditions. The degree of anesthesia was considered sufficient when, after a mechanical pressing on one of the animal’s hind limbs, no flexion reflex was observed. After the induction of anesthesia, mice were placed and their heads were stabilized at three points by two blunt ear bars and anterior bite bar in a stereotactic frame (51,900; Stoelting, Wood Dale, IL, USA) with an additional adaptor (51,625; Stoelting, Wood Dale, IL, USA). After trimming the hair using an electric shaver (9667L; 3 M Health Care, Saint Paul, MN, USA) and subsequent preparation of an aseptic surgical field, the longitudinal incision was made in the skin overlying the skull exposing both the sagittal and coronal sutures. The skull surface was then instilled with a 3% hydrogen peroxide solution (H_2_O_2_) to more precisely identify and visualize the bregma and lambda points and achieve hemostasis and disinfection. The animals’ eyes were additionally instilled with a 0.15% solution of sodium hyaluronate (C_28_H_44_N_2_O_23_ · Na) and 2% dexpanthenol (C_9_H_19_NO_4_) in the form of a ready-made solution (Bepanthen Eye; Bayer, Leverkusen, Germany) to obtain protection against excessive drying and damage to the cornea during the procedures. Using a set of micrometer screws, the entry points of the intracerebral cannula were marked on the skull vault, where the position of the needle was zeroed by reading the coordinates at the bregma point. The burr holes were made bilaterally using a sterile needle (4,665,112; B. Braun, Melsungen, Germany) 19G × 1½″ (∅ 1.1 × 40 mm) above the intended cannula insertion sites based on the scheduled stereotactic coordinates. Injection and infusion were performed using an automatic pump (UMP2; World Precision Instruments, Sarasota, FL, USA) equipped with a microsyringe (NanoFil-100; World Precision Instruments, Sarasota, FL, USA) and an appropriate needle (NF35BV‐2; World Precision Instruments, Sarasota, FL, USA) mounted on the movable arm of a stereotaxic table moving in three axes—antero-posterior (AP), medial–lateral (ML), and dorsal–ventral (DV)—connected with a programmable controller (Micro 4; World Precision Instruments, Sarasota, FL, USA). After visual identification of the dura, the microneedle was slowly introduced into the brain and then waited 2 min before starting the infusion to allow the brain to return to normal topography after transient mechanical deformation. A programmable microsyringe pump was used to deliver bilaterally 4 µL (8 μL per intact brain) of Atsttrin (Atreaon, Inc.; Newton, CA, USA) dissolved in a sterile 0.9% saline solution (NaCl) with doses of 0.1 μg (0.025 μg/μL), 0.5 μg (0.125 μg/μL), 1 μg (0.25 μg/μL), 2 μg (0.5 μg/μL), and 5 μg (1.25 μg/μL) into ST at the following stereotactic coordinates—AP (y), + 0.62; ML (x), ± 1.75 relative to the bregma; and DV (z), − 3.5 mm relative to the dura (Paxinos G, Franklin KBJ. The Mouse Brain in Stereotaxic Coordinates, 2nd Edition; Academic Press, 2001, San Diego, CA, USA) with automatic flow rate estimated as 0.5 μL/min [35]. After the injection, the microneedle was kept in the brain for 3 min to minimize the risk of reflux of the injected substance along the intracranial path of the withdrawn cannula. After this time, the microneedle was removed, the burr holes were filled with bone wax (060.196.0057; Atramat, Ciudad de México, Mexico) using a double-sided dissector, and the scalp wound was closed with single monofilament non-absorbable 5–0 nylon (polyamide) sutures using a needle in size 16 mm with a curvature of 3/8 of a circle (DK05PA; Yavo, Bełchatów, Poland). The wound area was finally disinfected with a 7.5% povidone-iodine solution (Braunol; B. Braun, Melsungen, Germany). Animals from the control groups were subjected to identical anesthesia and stereotaxic surgery involving a bilateral intracerebral injection of an equal volume of sterile Ringer’s solution containing 8.6 g/dm^3^ NaCl, 0.3 g/dm^3^ KCl and 0.33 g/dm^3^ CaCl_2_ · 2H_2_O (Fresenius Kabi, Warsaw, Poland). After the procedures, the animals were placed in cages with ad libitum access to water and food. In order to minimize the risk of significant hypothermia in animals during the perioperative period, an appropriately controlled high ambient temperature (28 ± 2 °C) was maintained.

### MPTP Intoxication

In order to induce a set of symptoms related to damage to the extrapyramidal system as well as subsequent biochemical and neuropathological alternations constituting a model imitating the changes occurring in Parkinson’s disease, intraperitoneal injections of MPTP neurotoxin (68,750; Sigma-Aldrich, St. Louis, MO, USA) were performed in mice [36]. In accordance with the study protocol, MPTP intoxication were performed after stereotactic operations covering Atsttrin or Ringer’s solution administration into ST at designated time points within appropriate groups of animals. Regarding the high toxicity (CAS#: 23,007–85-4), the compound was each time prepared under appropriate conditions and dissolved in a tightly closed marked ampoule in a sterile 0.9% NaCl solution (Polfa SA, Lublin, Poland) 5 min before the injection procedure began. The adopted administration scheme covered four serial intraperitoneal injections using a 25G × 5/8″ (∅ 0.5 × 16 mm) needle with a 1-mL syringe (300,014; BD Plastipak, Madrid, Spain) under appropriate aseptic conditions at 1-h intervals at a dose of 10 mg/kg body weight (volume 0.1 mL/20 g body weight) to a total dose of 40 mg/kg body weight (4 × 0.12 mL) of pure MPTP, which corresponded to 47 mg/kg of the MPTP hydrochloride (MPTP-HCl) [37]. The dose of MPTP used in study, adjusted according to the age group and sex of the animals covered in this case, was the most effective regimen, taking into account the decrease (≥ 80%) profile of DA concentration level within the nigrostriatal pathways [38]. The intoxications of MPTP were carried out in a special laboratory, between 10:00–15:00 (UTC + 01:00), each time by a suitably skilled person provided with appropriate personal protective equipment (safety goggles, FFP3 mask, protective suit, nitrile gloves, and shoe protectors) maintaining all safety procedures. In order to minimize the risk of significant hypothermia in animals during MPTP intoxication procedure, an appropriately controlled high ambient temperature (28 ± 2 °C) was maintained.

### Tissue Dissection and Preparation

At the assumed time points, in accordance with the study protocol, the animals were killed by dislocation of the cervical spine, causing a disruption of the continuity of the spinal cord. Decapitation was performed at the level of the cephalo-cervical joints (C_0_–C_1_/C_2_) using Mayo scissors. In the next stage, a long longitudinal linear cut of the skin was made, and the bones of the skull were separated along the sagittal suture, and the vault was successively resected by fragmenting it using microscissors. The intact brain was completely removed from the skull base bones and cranial nerves and then put on an ice-cold glass plate (10 × 15 × 0.5 cm). Using microsurgical technique and appropriate surgical tools under microscope control (SK-292H; Opta-Tech, Warsaw, Poland) samples of the ST, the hippocampus (CA), cerebral cortex (CX), and cerebellum cortex (CM) were dissected and obtained bilaterally. In the first stage of isolation, two preparations of the CX of the brain were obtained, and then, the structures located in the posterior cranial fossa were separated from the hemispheres, obtaining preparations of the CM. Next, an incision was made in the midline of the hemispheres, and the lateral ventricle of the brain was opened along the choroid fissure, dissecting the CA and then obtaining the gray matter of the ST. The collected brain tissue samples were then weighed (XS105 Dual Range; Mettler Toledo, Greifensee, Switzerland) and placed on dry ice (CO_2_) than frozen at − 80 °C until molecular and biochemical analyses were made. All dissection procedures were carried out in a highly reproducible manner so as to exclude any significant impact on the obtained results.

### Real-Time PCR Analysis

Extraction of total RNA from the ST, CA, CX, and CM was performed using a modified AGPC (guanidinium thiocyanate-phenol–chloroform) method developed by Chomczyński and Sacchi [39]. Total RNA was extracted from the obtained neuroanatomical tissue samples using the TRI Reagent (T9424; Sigma-Aldrich, St. Louis, MO, USA) according to the manufacturer standard protocol. The concentration of isolated RNA was quantified spectrophotometrically at 260 nm using BioPhotometerD30 (6,133,000,001; Eppendorf, Hamburg, Germany) and TrayCell (Z802573; Hellma GmbH, Müllheim, Germany). In order to obtain a single-stranded complementary DNA (cDNA), the previously isolated RNA was subjected to a reverse transcription (RT) reaction using PrimeScript RT Reagent (RR037A; Takara Bio, Otsu, Japan). Incubation was carried out in a SensoQuest Labcycler (011–101; SensoQuest GmbH, Göttingen, Germany) for 15 min at 37 °C and then at 85 °C for 5 s in order to denature potentially formed RNA/cDNA hybrids. The cDNA material thus obtained was stored at − 20 °C, and the amplification reaction was performed. Amplification of appropriate cDNA fragments was performed using real-time PCR with live monitoring and analysis of the kinetics of product growth. In this case, the Rotor-Gene Q 5plex HRM System (73070BC; Qiagen Benelux BV, Velno, Netherlands) with compatible Rotor-Gene Q Series Software (version 2.1.0; Qiagen Benelux BV, Velno, Netherlands) was used. The reaction was carried out using a mixture consisting of 1 μL of the previously obtained cDNA, 10 μL of FastStart Essential DNA Green Master (06402712001; Roche Molecular Systems, Alameda, CA, USA), 1.25 μL of “forward” primer (F), 1.25 μL of “reverse” primer (R), and 6.5 μL of nuclease-free water (06924204001; Roche Molecular Systems, Alameda, CA, USA). The cDNA was amplified with gene-specific primers designed using Primer BLAST software (http://www.ncbi.nlm.nih.gov/tools/primer-blast) of the National Center for Biotechnology Information (NCBI) database (Table 3). The housekeeping gene covering glyceraldehyde 3-phosphate dehydrogenase (GAPDH) was used to normalize gene expression levels. The amplification protocol was as follows: initial denaturation at 95 °C for 10 min, then 45 cycles covering denaturation at 95 °C for 15 s, attachment of primers at 58 °C for 15 s, and synthesis at 72 °C for 15 s. The reaction melting-curve analysis was applied to all reactions to ensure the consistency and specificity of the amplified product. Every sample and amplifications was analyzed in duplicate. The relative expression of the target genes was calculated by estimation according to the Pfaffl method [40].

**Table 3:** Sequences of primers used in real-time PCR reactions and expected lengths of the obtained products

Amplified gene	Primer sequences (5′ → 3′)		Predicted product size (bp)	GenBank reference sequence accession number (NCBI)
GAPDH	F	5′–TGCACCACCAACTGCTTAGC–3′	87	NM_001289726.1
	R	5′–GGCATGGACTGTGGTCATGAG–3′		
IL-1α	F	5′–ACGTCAAGCAACGGGAAGAT–3′	124	NM_010554.4
	R	5′–AAGGTGCTGATCTGGGTTGG–3′		
IL-6	F	5′–GAGGATACCACTCCCAACAGACC–3′	141	NM_031168.2
	R	5′–AAGTGCATCATCGTTGTTCATACA–3′		
TNFα	F	5′–AGCCGATGGGTTGTACCTTG–3′	99	NM_013693.3
	R	5′–ATAGCAAATCGGCTGACGGT–3′		
TH	F	5′–AACCTACCAGCCGGTGTACT–3′	94	NM_009377.2
	R	5′–AGAGAATGGGCGCTGGATAC–3′		
TG2	F	5′–TCAGCAAGTGAAGTACGGGC–3′	106	NM_009373.3
	R	5′–GGCGGAGTTGTAGTTGGTCA–3′		

*GAPDH* glyceraldehyde 3-phosphate dehydrogenase, *IL-1α* interleukin 1 alpha, *IL-6* interleukin 6, *TNFα* tumor necrosis factor alpha, *TH* tyrosine hydroxylase, *TG2* transglutaminase 2, *F* forward primer, *R* reverse primer

### HPLC Analysis

The examined ST, CA, CX, and CM concentrations of monoamines including DA, DOPAC, 3-MT, HVA, NA, MHPG, 5-HT, and 5-HIAA were determined using HPLC method. Before analysis, brain samples were homogenized using ultrasonic cell disrupter (VirSonic 60; VirTis, Gardiner, NY, USA) in 1000 μL of homogenization mixture containing ice-cold 0.1 M perchloric acid (HClO_4_) and 0.05 mM ascorbic acid (C_6_H_8_O_6_). The samples were successively centrifuged for 15 min with 13.000 × g rate (Labofuge 400R; Heraeus Instruments, Hanau, Germany) at 4 °C. After filtration using syringe polytetrafluoroethylene (PTFE) membrane filters with pore size 0.2 μm (6792–1302 Puradisc; Whatman, UK), the supernatant (20 µL) was collected and placed in the HPLC apparatus with electrochemical detection (HPLC-ED) system. The HPLC system consisted of an automatic autosampler injector (LaChrom L-7250; Merck-Hitachi, Darmstadt/Tokio, Germany/Japan), pump (Mini-Star K-500; Knauer, Berlin, Germany), electrochemical detector (L-3500A; Merck-Recipe, Darmstadt/Monachium, Germany) with a glassy-carbon working electrode. The mobile phase (eluent) comprised a citrate–phosphate buffer solution of 32 mM sodium phosphate (NaH_2_PO_4_; Sigma-Aldrich, St. Louis, MO, USA), 34 mM citric acid (C_6_H_8_O_7_; Sigma-Aldrich, St. Louis, MO, USA), 1 mM octane sulfonic acid (C_8_H_18_O_3_S; Sigma-Aldrich, St. Louis, MO, USA), and 54 µM ethylenediaminetetraacetic acid (EDTA; Sigma-Aldrich, St. Louis, MO, USA) buffer in ultrapure water (18.3 MΩ ∙ cm) containing 16% methanol (CH_3_OH; Merck, Darmstadt, Germany). Monoamines were separated isocratically using EC 250/4 Nucleosil 100–5 C18AB (720,936.40; Macherey–Nagel, Düren, Germany) with dimensions of 250 mm × 4 mm with an average particle size of 5 µm and a pore size of 100 Å at a flow of 0.8 mL/min and an appropriate electrochemical potential of + 0.8 V relative to the silver/silver chloride (Ag/AgCl) electrode. The chromatograms were recorded and integrated by the use of the computerized data acquisition Clarity software (version 5.0; DataApex, Prague, Czech Republic). The monoamine concentrations were quantified and calculated by comparison with the standard reference solutions (external calibration). The monoamine concentrations in the sample were expressed as picogram per milligram wet tissue.

### Statistical Analysis

The data were analyzed using the Python (version 3.9.15; Python Software Foundation, Wilmington, DE, USA) and Jupyter Notebook (version 6.5.2; Project Jupyter, San Francisco, CA, USA) software package for Windows (Microsoft Corporation, Redmond, WA, USA). For this purpose, individual modules and sets of numerical algorithms included in the SciPy (version 1.9.3), Matplotlib (version 3.6.2), NumPy (version 1.23.5) oraz Pandas (version 1.5.2) libraries were used. For complementary analyses and comparative calculations, a Microsoft Office Excel 2010 software package for Windows (Microsoft Corporation, Redmond, WA, USA) was additionally used. The scipy.stats.ttest_ind function was used to assess differences between individual groups using a two-tailed Welch’s *t*-test, which does not assume equality of variances in the study and control populations and assumes the alternative hypothesis that the means of distribution in the study and control populations are not equal. The results were considered statistically significant when *p* values were less than 0.05 (*p* < 0.05). Histograms were used to present the general characteristics of the empirical distribution of the obtained results. The results were presented in bar charts as mean values ± standard error (SEM).

## Results

### Effect of the Increasing Doses of Atsttrin on the mRNA Expression Level of Selected Mediators and Enzymes

#### Evaluation and Assessment of the IL-1α Expression Level

**Fig. 3:**
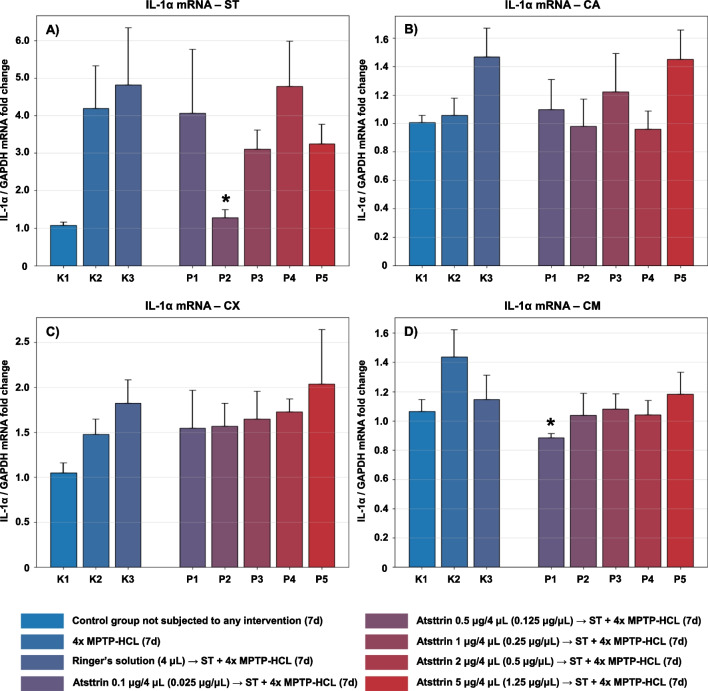
Changes in the expression of mRNA for IL-1α assessed after intracerebral administration into ST of five increasing doses of Atsttrin using stereotactic methods in C57BL/6 mice subjected to MPTP intoxication within ST (**A**), CA (**B**), CX (**C**), and CM (**D**). The results were expressed in the form of a semi-quantitative analysis as the fluorescence value of the test sample in relation to the fluorescence of the reference gene GAPDH (fold change). The results were presented as mean values ± SEM. Asterisk (*), difference from the appropriate control group (K2), *p < 0.05, **p < 0.01, ***p < 0.001; number sign (#), difference from the appropriate control group (K3), ^#^*p* < 0.05; ^##^*p* < 0.01; ^###^*p* < 0.001

The expression level of IL-1α mRNA within selected neuroanatomical structures in male C57BL/6 mice after MPTP intoxication and intracerebral stereotactic administration of five increasing Atsttrin doses into the ST was analyzed using real-time PCR method (Fig. 3). Intraperitoneal intoxication with MPTP in C57BL/6 mice was associated with an overall mean increase of IL-1α mRNA expression levels within all evaluated neuroanatomical structures among the control groups (K2–K3). Intracerebral stereotaxic administration of Atsttrin into the ST at a dose of 0.5 μg (0.125 μg/μL)/P2 in animals subjected to MPTP intoxication was associated with a statistically significant (*p* = 0.0383) decrease of the IL-1α mRNA expression level within the ST compared to the control group (K2). Moreover, intracerebral stereotaxic administration of lower (P1) and subsequently higher (P3–P5) doses of Atsttrin into the ST in animals after MPTP intoxication was not associated with a statistically significant change in the IL-1α mRNA expression level in the ST compared to the control groups (K2–K3). There were no statistically significant differences in the IL-1α mRNA expression level within the CA and CX in animals after MPTP-HCL intoxication and intracerebral stereotaxic administration of five increasing Atsttrin doses into the ST compared to the control groups (K2–K3). Intracerebral stereotaxic administration of Atsttrin into the ST at a dose of 0.1 μg (0.025 μg/μL)/P1 in animals subjected to MPTP intoxication was associated with a statistically significant (*p* = 0.0213) decrease of the IL-1α mRNA expression level within the CM compared to the control group (K2). Moreover, intracerebral stereotaxic administration of subsequently higher (P2–P5) doses of Atsttrin into the ST in animals after MPTP intoxication was not associated with a statistically significant change in the IL-1α mRNA expression level in the CM compared to the control groups (K2–K3).

#### Evaluation and Assessment of the TNFα Expression Level

**Fig. 4:**
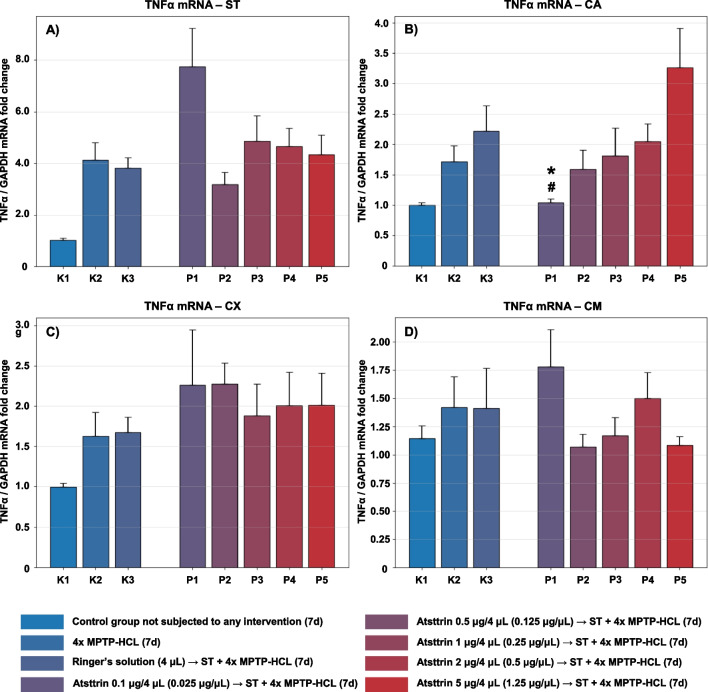
Changes in the expression of mRNA for TNFα assessed after intracerebral administration into ST of five increasing doses of Atsttrin using stereotactic methods in C57BL/6 mice subjected to MPTP intoxication within ST (**A**), CA (**B**), CX (**C**), and CM (**D**). The results were expressed in the form of a semi-quantitative analysis as the fluorescence value of the test sample in relation to the fluorescence of the reference gene GAPDH (fold change). The results were presented as mean values ± SEM. Asterisk (*), difference from the appropriate control group (K2), *p < 0.05, **p < 0.01, ***p < 0.001; Number sign (#), difference from the appropriate control group (K3), ^#^*p* < 0.05; ^##^*p* < 0.01; ^###^*p* < 0.001

The expression level of TNFα mRNA within selected neuroanatomical structures in male C57BL/6 mice after MPTP intoxication and intracerebral stereotactic administration of five increasing Atsttrin doses into the ST was analyzed using real-time PCR method (Fig. 4). Intraperitoneal intoxication with MPTP in C57BL/6 mice was associated with an overall mean increase of TNFα mRNA expression levels within all evaluated neuroanatomical structures among the control groups (K2–K3). There were no statistically significant differences in the TNFα mRNA expression level within the ST in animals after MPTP intoxication and intracerebral stereotaxic administration of five increasing Atsttrin doses into the ST compared to the control groups (K2–K3). Intracerebral stereotaxic administration of Atsttrin into the ST at a dose of 0.1 μg (0.025 μg/μL)/P1 in animals subjected to MPTP intoxication was associated with a statistically significant (*p* = 0.0327/*p* = 0.0465) decrease of the TNFα mRNA expression level within the CA compared to the control groups (K2/K3). Moreover, intracerebral stereotaxic administration of subsequently higher (P2–P5) doses of Atsttrin into the ST in animals after MPTP intoxication was not associated with a statistically significant change in the TNFα mRNA expression level in the CA compared to the control groups (K2–K3). There were no statistically significant differences in the TNFα mRNA expression level within the CX and CM in animals after MPTP-HCL intoxication and intracerebral stereotaxic administration of five increasing Atsttrin doses into the ST compared to the control groups (K2–K3).

#### Evaluation and Assessment of the IL-6 Expression Level

**Fig. 5:**
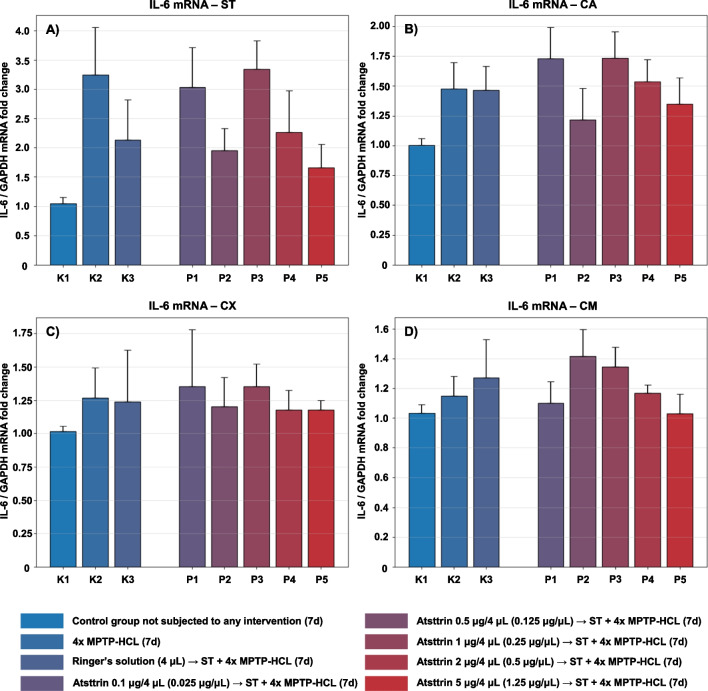
Changes in the expression of mRNA for IL-6 assessed after intracerebral administration into ST of five increasing doses of Atsttrin using stereotactic methods in C57BL/6 mice subjected to MPTP intoxication within ST (**A**), CA (**B**), CX (**C**), and CM (**D**). The results were expressed in the form of a semi-quantitative analysis as the fluorescence value of the test sample in relation to the fluorescence of the reference gene GAPDH (fold change). The results were presented as mean values ± SEM. Asterisk (*), difference from the appropriate control group (K2), *p < 0.05, **p < 0.01, ***p < 0.001; Number sign (#), difference from the appropriate control group (K3), ^#^*p* < 0.05; ^##^*p* < 0.01; ^###^*p* < 0.001

The expression level of IL-6 mRNA within selected neuroanatomical structures in male C57BL/6 mice after MPTP intoxication and intracerebral stereotactic administration of five increasing Atsttrin doses into the ST was analyzed using real-time PCR method (Fig. 5). Intraperitoneal intoxication with MPTP in C57BL/6 mice was associated with an overall mean increase of IL-6 mRNA expression levels within all evaluated neuroanatomical structures among the control groups (K2–K3). There were no statistically significant differences in the IL-6 mRNA expression level within the ST, CA, CX, and CM in animals after MPTP-HCL intoxication and intracerebral stereotaxic administration of five increasing Atsttrin doses into the ST compared to the control groups (K2–K3).

#### Evaluation and Assessment of the TH Expression Level

**Fig. 6:**
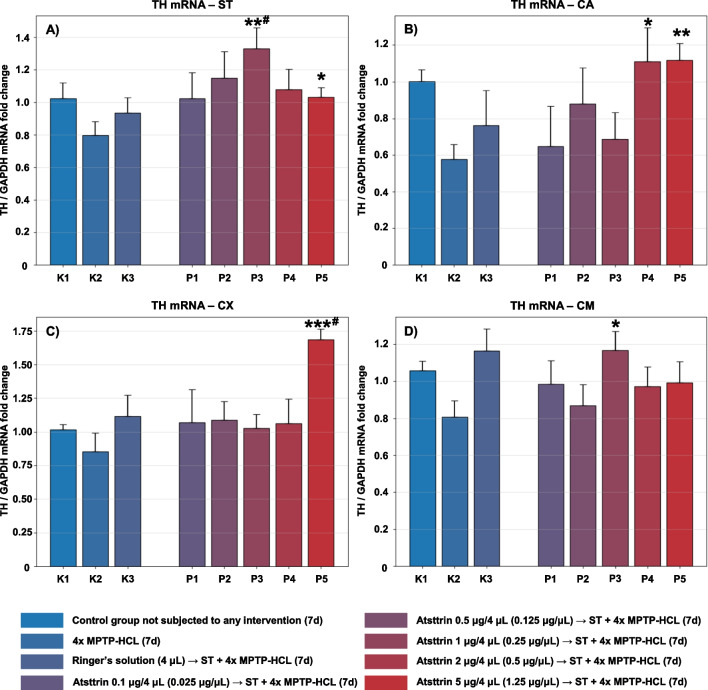
Changes in the expression of mRNA for TH assessed after intracerebral administration into ST of five increasing doses of Atsttrin using stereotactic methods in C57BL/6 mice subjected to MPTP intoxication within ST (**A**), CA (**B**), CX (**C**), and CM (**D**). The results were expressed in the form of a semi-quantitative analysis as the fluorescence value of the test sample in relation to the fluorescence of the reference gene GAPDH (fold change). The results were presented as mean values ± SEM. Asterisk (*), difference from the appropriate control group (K2), *p < 0.05, **p < 0.01, ***p < 0.001; number sign (#), difference from the appropriate control group (K3), ^#^*p* < 0.05; ^##^*p* < 0.01; ^###^*p* < 0.001

The expression level of TH mRNA within selected neuroanatomical structures in male C57BL/6 mice after MPTP intoxication and intracerebral stereotactic administration of five increasing Atsttrin doses into the ST was analyzed using real-time PCR method (Fig. 6). Intraperitoneal intoxication with MPTP in C57BL/6 mice was associated with an overall mean decrease of TH mRNA expression levels within all evaluated neuroanatomical structures among the control groups (K2–K3). Intracerebral stereotaxic administration of Atsttrin into the ST at a dose of 1 μg (0.25 μg/μL)/P3 in animals subjected to MPTP intoxication was associated with a statistically significant (*p* = 0.0072/*p* = 0.0367) increase of the TH mRNA expression level within the ST compared to the control groups (K2/K3). Furthermore, intracerebral stereotaxic administration of Atsttrin into the ST at a dose of 5 μg (1.25 μg/μL)/P5 in animals subjected to MPTP intoxication was associated with a statistically significant (*p* = 0.0420) increase of the TH mRNA expression level within the ST compared to the control group (K2). Intracerebral stereotaxic administration of Atsttrin into the ST at a dose of 2 μg (0.5 μg/μL)/P4 and 5 μg (1.25 μg/μL)/P5 in animals subjected to MPTP intoxication was associated with a statistically significant (*p* = 0.0421/*p* = 0.0010) increase of the TH mRNA expression level within the CA compared to the control groups (K2). Moreover, intracerebral stereotaxic administration of subsequently lower (P1–P3) doses of Atsttrin into the ST in animals after MPTP intoxication was not associated with a statistically significant change in the TH mRNA expression level in the CA compared to the control groups (K2–K3). Intracerebral stereotaxic administration of Atsttrin into the ST at a dose of 5 μg (1.25 μg/μL)/P5 in animals subjected to MPTP intoxication was associated with a statistically significant (*p* = 0.0003/*p* = 0.0169) increase of the TH mRNA expression level within the CX compared to the control groups (K2/K3). Moreover, intracerebral stereotaxic administration of subsequently lower (P1–P4) doses of Atsttrin into the ST in animals after MPTP intoxication was not associated with a statistically significant change in the TH mRNA expression level in the CX compared to the control groups (K2–K3). Intracerebral stereotaxic administration of Atsttrin into the ST at a dose of 1 μg (0.25 μg/μL)/P3 in animals subjected to MPTP intoxication was associated with a statistically significant (*p* = 0.0235) increase of the TH mRNA expression level within the CM compared to the control groups (K2). Moreover, intracerebral stereotaxic administration of subsequently lower (P1–P2) and higher (P4–P5) doses of Atsttrin into the ST in animals after MPTP intoxication was not associated with a statistically significant change in the TH mRNA expression level in the CM compared to the control groups (K2–K3).

#### Evaluation and Assessment of the TG2 Expression Level

**Fig. 7:**
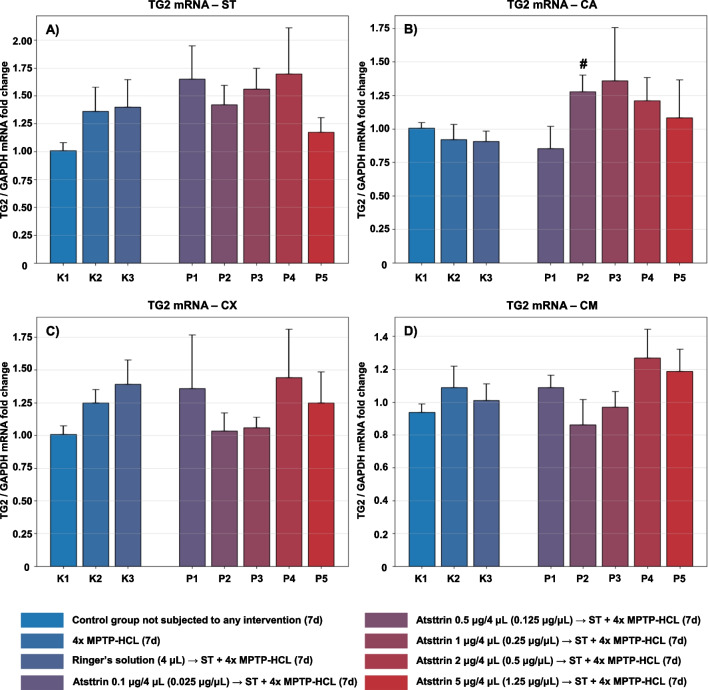
Changes in the expression of mRNA for TG2 assessed after intracerebral administration into ST of five increasing doses of Atsttrin using stereotactic methods in C57BL/6 mice subjected to MPTP intoxication within ST (**A**), CA (**B**), CX (**C**), and CM (**D**). The results were expressed in the form of a semi-quantitative analysis as the fluorescence value of the test sample in relation to the fluorescence of the reference gene GAPDH (fold change). The results were presented as mean values ± SEM. Asterisk (*), difference from the appropriate control group (K2), *p < 0.05, **p < 0.01, ***p < 0.001; number sign (#), difference from the appropriate control group (K3), ^#^*p* < 0.05; ^##^*p* < 0.01; ^###^*p* < 0.001

The expression level of TG2 mRNA within selected neuroanatomical structures in male C57BL/6 mice after MPTP intoxication and intracerebral stereotactic administration of five increasing Atsttrin doses into the ST was analyzed using real-time PCR method (Fig. 7). Intraperitoneal intoxication with MPTP in C57BL/6 mice was associated with an overall mean increase of TG2 mRNA expression levels within the majority of the evaluated neuroanatomical structures among the control groups (K2–K3). There were no statistically significant differences in the TG2 mRNA expression level within the ST in animals after MPTP intoxication and intracerebral stereotaxic administration of five increasing Atsttrin doses into the ST compared to the control groups (K2–K3). Intracerebral stereotaxic administration of Atsttrin into the ST at a dose of 0.5 μg (0.125 μg/μL)/P2 in animals subjected to MPTP intoxication was associated with a statistically significant (*p* = 0.0307) increase of the TG2 mRNA expression level within the CA compared to the control group (K3). Moreover, intracerebral stereotaxic administration of subsequently lower (P1) and higher (P3–P5) doses of Atsttrin into the ST in animals after MPTP intoxication was not associated with a statistically significant change of the TG2 mRNA expression level in the CA compared to the control groups (K2–K3). There were no statistically significant differences in the TG2 mRNA expression level within the CX and CM in animals after MPTP-HCL intoxication and intracerebral stereotaxic administration of five increasing Atsttrin doses into the ST compared to the control groups (K2–K3).

### Effect of Increasing Doses of Atsttrin on the Concentration Level of Monoamines

#### Evaluation and Assessment of the Dopaminergic System

**Fig. 8:**
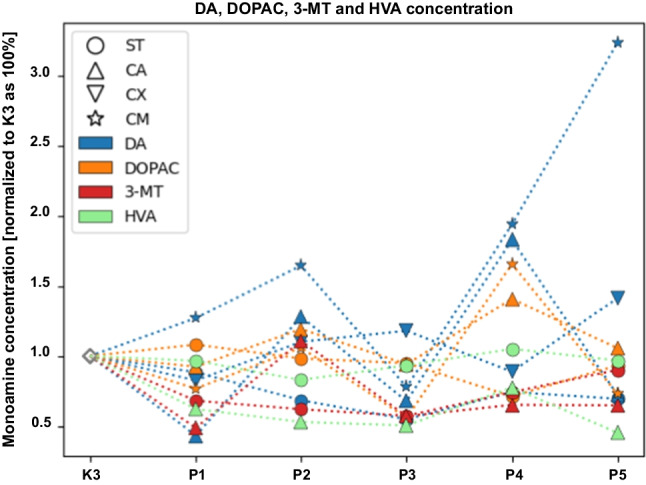
Changes in the concentration of DA, DOPAC, 3-MT, and HVA assessed after intracerebral administration into ST of five increasing doses of Atsttrin using stereotactic methods in C57BL/6 mice subjected to MPTP intoxication within ST, CA, CX, and CM. The monoamine concentration was normalized to control group (K3) as 100% (pg/mg)

**Fig. 9:**
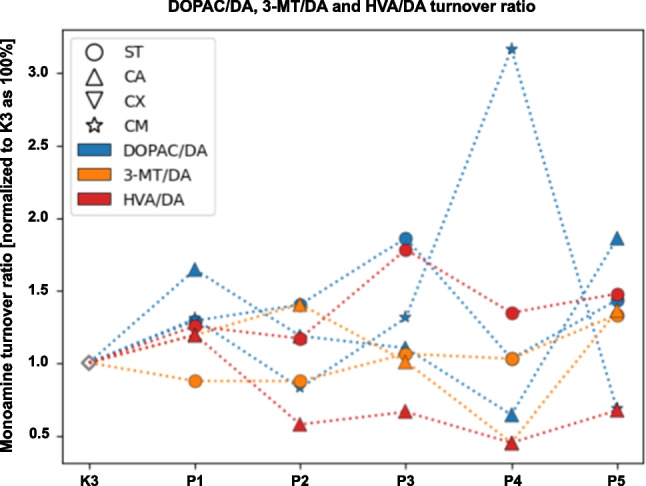
Changes in the turnover ratio of DOPAC/DA, 3-MT/DA, and HVA/DA assessed after intracerebral administration into ST of five increasing doses of Atsttrin using stereotactic methods in C57BL/6 mice subjected to MPTP intoxication within ST, CA, CX, and CM. The monoamine turnover ratio was normalized to the control group (K3) as 100% (pg/mg)

The concentration level of monoamines of the dopaminergic system within selected neuroanatomical structures in male C57BL/6 mice after MPTP intoxication and intracerebral stereotactic administration of five increasing Atsttrin doses into the ST was analyzed using the HPLC method (Fig. 8). Intraperitoneal intoxication with MPTP in C57BL/6 mice was associated with an overall mean decrease of DA concentration levels within the majority of the evaluated neuroanatomical structures among the control groups (K2–K3). Intracerebral stereotaxic administration of Atsttrin into the ST at a dose of 0.5 μg (0.125 μg/μL)/P2 in animals subjected to MPTP intoxication was associated with a statistically significant (*p* = 0.0481) increase of the DA concentration level within the ST compared to the control group (K2). Furthermore, intracerebral stereotaxic administration of Atsttrin into the ST at a dose of 1 μg (0.25 μg/μL)/P3 in animals subjected to MPTP intoxication was associated with a statistically significant (*p* = 0.0155/*p* = 0.0499) increase of the DA concentration level within the ST compared to the control group (K2/K3). There were no statistically significant differences in the DA concentration level within the CA, CX, and CM in animals after MPTP-HCL intoxication and intracerebral stereotaxic administration of five increasing Atsttrin doses into the ST compared to the control groups (K2–K3). Detailed statistics of the DA concentration levels can be found in Supplementary Fig. 1. Intraperitoneal intoxication with MPTP in C57BL/6 mice was associated with an overall mean increase of DOPAC concentration levels within the majority of the evaluated neuroanatomical structures among the control groups (K2–K3). Intracerebral stereotaxic administration of Atsttrin into the ST at a dose of 2 μg (0.5 μg/μL)/P4 in animals subjected to MPTP intoxication was associated with a statistically significant (*p* = 0.0148) decrease of the DOPAC concentration level within the ST compared to the control group (K2). Moreover, intracerebral stereotaxic administration of subsequently lower (P1–P3) and higher (P5) doses of Atsttrin into the ST in animals after MPTP intoxication was not associated with a statistically significant change of the DOPAC concentration level in the ST compared to the control groups (K2–K3). There were no statistically significant differences in the DOPAC concentration level within the CA and CX in animals after MPTP-HCL intoxication and intracerebral stereotaxic administration of five increasing Atsttrin doses into the ST compared to the control groups (K2–K3). Intracerebral stereotaxic administration of Atsttrin into the ST at a dose of 0.1 μg (0.025 μg/μL)/P1, 1 μg (0.25 μg/μL)/P3, and 5 μg (1.25 μg/μL)/P5 in animals subjected to MPTP intoxication was associated with a statistically significant (*p* = 0.0193/*p* = 0.0057/*p* = 0.0116) decrease of the DOPAC concentration level within the CM compared to the control group (K2). Detailed statistics of the DOPAC concentration levels can be found in Supplementary Fig. 2. Intraperitoneal intoxication with MPTP in C57BL/6 mice was associated with various changes of 3-MT concentration levels within the evaluated neuroanatomical structures among the control groups (K2–K3). Intracerebral stereotaxic administration of Atsttrin into the ST at a dose of 0.5 μg (0.125 μg/μL)/P2 and 1 μg (0.25 μg/μL)/P3 in animals subjected to MPTP intoxication was associated with a statistically significant (*p* = 0.0311/*p* = 0.0157) decrease of the 3-MT concentration level within the ST compared to the control group (K2). Furthermore, intracerebral stereotaxic administration of Atsttrin into the ST at a dose of 1 μg (0.25 μg/μL)/P3 in animals subjected to MPTP intoxication was associated with a statistically significant (*p* = 0.0348) decrease of the 3-MT concentration level within the ST compared to the control group (K3). There were no statistically significant differences in the 3-MT concentration level within the CA in animals after MPTP-HCL intoxication and intracerebral stereotaxic administration of five increasing Atsttrin doses into the ST compared to the control groups (K2–K3). Intracerebral stereotaxic administration of Atsttrin into the ST at a dose 0.1 μg (0.025 μg/μL)/P1, 1 μg (0.25 μg/μL)/P3, 2 μg (0.5 μg/μL)/P4, and 5 μg (1.25 μg/μL)/P5 in animals subjected to MPTP intoxication was associated with a statistically significant (*p* = 0.0001/*p* = 0.0017/*p* = 0.0085/*p* = 0.0024) decrease of the 3-MT concentration level within the CX compared to the control group (K2). There were no statistically significant differences in the 3-MT concentration level within the CM in animals after MPTP intoxication and intracerebral stereotaxic administration of five increasing Atsttrin doses into the ST compared to the control groups (K2–K3). Detailed statistics of the 3-MT concentration levels can be found in Supplementary Fig. 3. Intraperitoneal intoxication with MPTP in C57BL/6 mice was associated with an overall mean decrease of HVA concentration levels within the majority of the evaluated neuroanatomical structures among the control groups (K2–K3). There were no statistically significant differences in the HVA concentration level within the ST and CA in animals after MPTP-HCL intoxication and intracerebral stereotaxic administration of five increasing Atsttrin doses into the ST compared to the control groups (K2–K3). Intracerebral stereotaxic administration of Atsttrin into the ST at a dose 0.5 μg (0.125 μg/μL)/P2, 1 μg (0.25 μg/μL)/P3, and 5 μg (1.25 μg/μL)/P5 in animals subjected to MPTP intoxication was associated with a statistically significant (*p* = 0.0060/*p* = 0.0472/*p* = 0.0284) decrease of the HVA concentration level within the CX compared to the control group (K3). There were no statistically significant differences in the HVA concentration level within the CM in animals after MPTP intoxication and intracerebral stereotaxic administration of five increasing Atsttrin doses into the ST compared to the control groups (K2–K3). Detailed statistics of the HVA concentration levels can be found in Supplementary Fig. 4. The turnover ratios of DOPAC/DA, 3-MT/DA, and HVA/DA within selected neuroanatomical structures in male C57BL/6 mice after MPTP intoxication and intracerebral stereotactic administration of five increasing Atsttrin doses into the ST were estimated basing on the ratio of concentration levels of DA,DOPAC, 3-MT, and HVA analyzed by HPLC method (Fig. 9). Intraperitoneal intoxication with MPTP in C57BL/6 mice was associated with an overall mean increase of DOPAC/DA turnover ratios within all evaluated neuroanatomical structures among the control groups (K2–K3). Intracerebral stereotaxic administration of Atsttrin into the ST at a dose of 0.5 μg (0.125 μg/μL)/P2, 1 μg (0.25 μg/μL)/P3, and 5 μg (1.25 μg/μL)/P5 in animals subjected to MPTP intoxication was associated with a statistically significant (*p* = 0.0323/*p* = 0.0148/*p* = 0.0346) increase of the DOPAC/DA turnover ratio within the ST compared to the control group (K3). There were no statistically significant differences in the DOPAC/DA turnover ratios within the CA, CX, and CM in animals after MPTP intoxication and intracerebral stereotaxic administration of five increasing Atsttrin doses into the ST compared to the control groups (K2–K3). Detailed statistics of the DOPAC/DA turnover ratios can be found in Supplementary Fig. 5. Intraperitoneal intoxication with MPTP in C57BL/6 mice was associated with an overall mean increase of 3-MT/DA turnover ratios within all evaluated neuroanatomical structures among the control groups (K2–K3). Intracerebral stereotaxic administration of Atsttrin into the ST at a dose of 5 μg (1.25 μg/μL)/P5 in animals subjected to MPTP intoxication was associated with a statistically significant (*p* = 0.0206) increase of the 3-MT/DA turnover ratio within the ST compared to the control group (K2). Moreover, intracerebral stereotaxic administration of subsequently lower (P1–P4) doses of Atsttrin into the ST in animals after MPTP intoxication was not associated with a statistically significant change in the 3-MT/DA turnover ratio in the ST compared to the control groups (K2–K3). There were no statistically significant differences in the 3-MT/DA turnover ratio within the CA in animals after MPTP intoxication and intracerebral stereotaxic administration of five increasing Atsttrin doses into the ST compared to the control groups (K2–K3). Intracerebral stereotaxic administration of Atsttrin into the ST at a dose of 2 μg (0.5 μg/μL)/P4 in animals subjected to MPTP intoxication was associated with a statistically significant (*p* = 0.0087) decrease of the 3-MT/DA turnover ratio within the CX compared to the control group (K2). Moreover, intracerebral stereotaxic administration of subsequently lower (P1–P3) and higher (P5) doses of Atsttrin into the ST in animals after MPTP intoxication was not associated with a statistically significant change in the 3-MT/DA turnover ratio in the CX compared to the control groups (K2–K3). There were no statistically significant difference in the 3-MT/DA turnover ratio within the CM in animals after MPTP intoxication and intracerebral stereotaxic administration of five increasing Atsttrin doses into the ST compared to the control groups (K2–K3). Detailed statistics of the 3-MT/DA turnover ratios can be found in Supplementary Fig. 6. Intraperitoneal intoxication with MPTP in C57BL/6 mice was associated with various changes of HVA/DA turnover ratios within all evaluated neuroanatomical structures among the control groups (K2–K3). Intracerebral stereotaxic administration of Atsttrin into the ST at a dose of 1 μg (0.25 μg/μL)/P3 and 2 μg (0.5 μg/μL)/P4 in animals subjected to MPTP intoxication was associated with a statistically significant (*p* = 0.0259/*p* = 0.0222) increase of the HVA/DA turnover ratio within the ST compared to the control group (K2). Furthermore, intracerebral stereotaxic administration of Atsttrin into the ST at a dose of 1 μg (0.25 μg/μL)/P3 in animals subjected to MPTP intoxication was associated with a statistically significant (*p* = 0.0381) increase of the HVA/DA turnover ratio within the ST compared to the control group (K3). There were no statistically significant differences in the HVA/DA turnover ratio within the CA in animals after MPTP intoxication and intracerebral stereotaxic administration of five increasing Atsttrin doses into the ST compared to the control groups (K2–K3). Intracerebral stereotaxic administration of Atsttrin into the ST at a dose of 2 μg (0.5 μg/μL)/P4 in animals subjected to MPTP intoxication was associated with a statistically significant (*p* = 0.0411) increase of the HVA/DA turnover ratio within the CX compared to the control group (K3). Moreover, intracerebral stereotaxic administration of subsequently lower (P1–P3) and higher (P5) doses of Atsttrin into the ST in animals after MPTP intoxication was not associated with a statistically significant change in the HVA/DA turnover ratio in the CX compared to the control groups (K2–K3). There were no statistically significant differences in the HVA/DA turnover ratio within the CM in animals after MPTP intoxication and intracerebral stereotaxic administration of five increasing Atsttrin doses into the ST compared to the control groups (K2–K3). Detailed statistics of the 3-MT/DA turnover ratios can be found in Supplementary Fig. 7.

#### Evaluation and Assessment of the Noradrenergic System

**Fig. 10:**
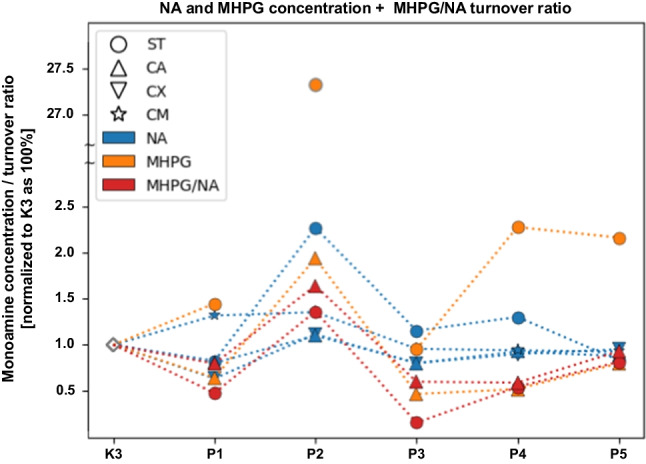
Changes in the concentration of NA and MHPG including MHPG/NA turnover ratio assessed after intracerebral administration into ST of five increasing doses of Atsttrin using stereotactic methods in C57BL/6 mice subjected to MPTP intoxication within ST, CA, CX, and CM. The monoamine concentration and turnover level were normalized in the control group (K3) as 100% (pg/mg)

The concentration level of monoamines of the noradrenergic system within selected neuroanatomical structures in male C57BL/6 mice after MPTP intoxication and intracerebral stereotactic administration of five increasing Atsttrin doses into the ST was analyzed using HPLC method (Fig. 10). Intraperitoneal intoxication with MPTP in C57BL/6 mice was associated with an overall mean decrease of NA concentration levels within all of the evaluated neuroanatomical structures among the control groups (K2-K3). There were no statistically significant differences in the NA concentration level within the ST in animals after MPTP intoxication and intracerebral stereotaxic administration of five increasing Atsttrin doses into the ST compared to the control groups (K2–K3). Intracerebral stereotaxic administration of Atsttrin into the ST at a dose of 0.1 μg (0.025 μg/μL)/P1 in animals subjected to MPTP intoxication was associated with a statistically significant (*p* = 0.0156) decrease of the NA concentration level within the CA compared to the control group (K2). Moreover, intracerebral stereotaxic administration of subsequently higher (P2–P5) doses of Atsttrin into the ST in animals after MPTP intoxication was not associated with a statistically significant change of the NA concentration level in the CA compared to the control groups (K2–K3). Intracerebral stereotaxic administration of Atsttrin into the ST at a dose of 0.1 μg (0.025 μg/μL)/P1 in animals subjected to MPTP intoxication was associated with a statistically significant (*p* = 0.0427) decrease of the NA concentration level within the CX compared to the control group (K3). Moreover, intracerebral stereotaxic administration of subsequently higher (P2–P5) doses of Atsttrin into the ST in animals after MPTP intoxication was not associated with a statistically significant change of the NA concentration level in the CX compared to the control groups (K2–K3). Intracerebral stereotaxic administration of Atsttrin into the ST at a dose of 1 μg (0.25 μg/μL)/P3, 2 μg (0.5 μg/μL)/P4, and 5 μg (1.25 μg/μL)/P5 in animals subjected to MPTP intoxication was associated with a statistically significant (*p* = 0.0103/*p* = 0.0127/*p* = 0.0091) decrease of the NA concentration level within the CM compared to the control group (K2). Furthermore, intracerebral stereotaxic administration of Atsttrin into the ST at a dose of 0.1 μg (0.025 μg/μL)/P1 and 0.5 μg (0.125 μg/μL)/P2 in animals subjected to MPTP intoxication was associated with a statistically significant (*p* = 0.0274/*p* = 0.0211) increase of the NA concentration level within the CM compared to the control group (K3). Detailed statistics of the NA concentration levels can be found in Supplementary Fig. 8. Intraperitoneal intoxication with MPTP in C57BL/6 mice was associated with an overall mean increase of MHPG concentration levels within all of the evaluated neuroanatomical structures among the control groups (K2–K3). There were no statistically significant differences in the MHPG concentration level within the ST and CA in animals after MPTP intoxication and intracerebral stereotaxic administration of five increasing Atsttrin doses into the ST compared to the control groups (K2–K3). Intracerebral stereotaxic administration of Atsttrin into the ST at a dose of 1 μg (0.25 μg/μL)/P3 and 2 μg (0.5 μg/μL)/P4 in animals subjected to MPTP intoxication was associated with a statistically significant (*p* = 0.0237/*p* = 0.0307) decrease of the MHPG concentration level within the CX compared to the control group (K3). There were no statistically significant differences in the MHPG concentration level within the CM in animals after MPTP intoxication and intracerebral stereotaxic administration of five increasing Atsttrin doses into the ST compared to the control groups (K2–K3). Detailed statistics of the MHPG concentration levels can be found in Supplementary Fig. 9. The turnover ratio of MHPG/NA within selected neuroanatomical structures in male C57BL/6 mice after MPTP intoxication and intracerebral stereotactic administration of five increasing Atsttrin doses into the ST was estimated basing on the ratio of concentration levels of NA and MHPG analyzed by HPLC method. Intraperitoneal intoxication with MPTP in C57BL/6 mice was associated with an overall mean increase of MHPG/NA turnover ratios within the majority of the evaluated neuroanatomical structures among the control groups (K2–K3). Intracerebral stereotaxic administration of Atsttrin into the ST at a dose of 1 μg (0.25 μg/μL)/P3 in animals subjected to MPTP intoxication was associated with a statistically significant (*p* = 0.0191) decrease of the MHPG/NA turnover ratio within the ST compared to the control group (K2). Moreover, intracerebral stereotaxic administration of subsequently lower (P1–P2) and higher (P4–P5) doses of Atsttrin into the ST in animals after MPTP intoxication was not associated with a statistically significant change in the MHPG/NA turnover ratio in the ST compared to the control groups (K2–K3). There were no statistically significant differences in the MHPG/NA turnover ratio within the CA in animals after MPTP intoxication and intracerebral stereotaxic administration of five increasing Atsttrin doses into the ST compared to the control groups (K2–K3). Intracerebral stereotaxic administration of Atsttrin into the ST at a dose of 1 μg (0.25 μg/μL)/P3 and 2 μg (0.5 μg/μL)/P4 in animals subjected to MPTP intoxication was associated with a statistically significant (*p* = 0.0353/*p* = 0.0434) decrease of the MHPG/NA turnover ratio within the CX compared to the control group (K3). There were no statistically significant differences in the MHPG/NA turnover ratio within the CM in animals after MPTP intoxication and intracerebral stereotaxic administration of five increasing Atsttrin doses into the ST compared to the control groups (K2–K3). Detailed statistics of the MHPG/NA turnover ratios can be found in Supplementary Fig. 10.

#### Evaluation and Assessment of the Serotonergic System

**Fig. 11:**
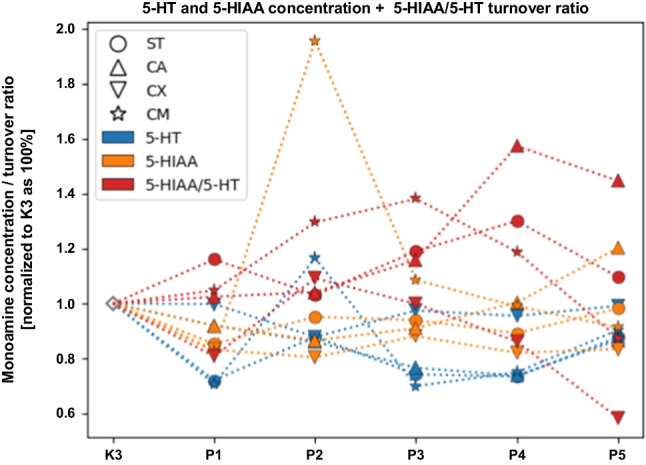
Changes in the concentration of 5-HT and 5-HIAA including 5-HIAA/5-HT turnover ratio assessed after intracerebral administration into ST of five increasing doses of Atsttrin using stereotactic methods in C57BL/6 mice subjected to MPTP intoxication within ST, CA, CX, and CM. The monoamine concentration and turnover level were normalized in the control group (K3) as 100% (pg/mg)

The concentration level of monoamines of the serotonergic system within selected neuroanatomical structures in male C57BL/6 mice after MPTP intoxication and intracerebral stereotactic administration of five increasing Atsttrin doses into the ST was analyzed using HPLC method (Fig. 11). Intraperitoneal intoxication with MPTP in C57BL/6 mice was associated with an overall mean increase of 5-HT concentration levels within all of the evaluated neuroanatomical structures among the control groups (K2–K3). There were no statistically significant differences in the 5-HT concentration level within the ST, CA, and CX in animals after MPTP intoxication and intracerebral stereotaxic administration of five increasing Atsttrin doses into the ST compared to the control groups (K2–K3). Intracerebral stereotaxic administration of Atsttrin into the ST at a dose of 0.1 μg (0.025 μg/μL)/P1, 1 μg (0.25 μg/μL)/P3, and 2 μg (0.5 μg/μL)/P4 in animals subjected to MPTP intoxication was associated with a statistically significant (*p* = 0.0420/*p* = 0.0400/*p* = 0.0480) decrease of the 5-HT concentration level within the CM compared to the control group (K3). Detailed statistics of the 5-HT concentration levels can be found in Supplementary Fig. 11. Intraperitoneal intoxication with MPTP in C57BL/6 mice was associated with an overall mean increase of 5-HIAA concentration levels within all of the evaluated neuroanatomical structures among the control groups (K2–K3). There were no statistically significant differences in the 5-HIAA concentration level within the ST and CA in animals after MPTP intoxication and intracerebral stereotaxic administration of five increasing Atsttrin doses into the ST compared to the control groups (K2–K3). Intracerebral stereotaxic administration of Atsttrin into the ST at a dose of 2 μg (0.5 μg/μL)/P4 and 5 μg (1.25 μg/μL)/P5 in animals subjected to MPTP intoxication was associated with a statistically significant (*p* = 0.0406/*p* = 0.0007) increase of the 5-HIAA concentration level within the CX compared to the control group (K2). Moreover, intracerebral stereotaxic administration of subsequently lower (P1–P3) doses of Atsttrin into the ST in animals after MPTP intoxication was not associated with a statistically significant change of the 5-HIAA concentration level in the CX compared to the control groups (K2–K3). There were no statistically significant differences in the 5-HIAA concentration level within the CM in animals after MPTP intoxication and intracerebral stereotaxic administration of five increasing Atsttrin doses into the ST compared to the control groups (K2–K3). Detailed statistics of the 5-HT concentration levels can be found in Supplementary Fig. 12. The turnover ratio of 5-HIAA/5-HT within selected neuroanatomical structures in male C57BL/6 mice after MPTP intoxication and intracerebral stereotactic administration of five increasing Atsttrin doses into the ST was estimated basing on the ratio of concentrations levels of 5-HT and 5-HIAA analyzed by HPLC method. Intraperitoneal intoxication with MPTP in C57BL/6 mice was associated with an overall mean increase of 5-HIAA/5-HT turnover ratios within the majority of the evaluated neuroanatomical structures among the control groups (K2–K3). There were no statistically significant differences in the 5-HIAA/5-HT turnover ratios within the ST and CA in animals after MPTP intoxication and intracerebral stereotaxic administration of five increasing Atsttrin doses into the ST compared to the control groups (K2–K3). Intracerebral stereotaxic administration of Atsttrin into the ST at a dose of 5 μg (1.25 μg/μL)/P5 in animals subjected to MPTP intoxication was associated with a statistically significant (*p* = 0.0289) increase of the 5-HIAA/5-HT turnover ratio within the CX compared to the control group (K3). Moreover, intracerebral stereotaxic administration of subsequently lower (P1–P4) doses of Atsttrin into the ST in animals after MPTP intoxication was not associated with a statistically significant change in the 5-HIAA/5-HT turnover ratio in the CX compared to the control groups (K2–K3). There were no statistically significant differences in the 5-HIAA/5-HT turnover levels within the CM in animals after MPTP intoxication and intracerebral stereotaxic administration of five increasing Atsttrin doses into the ST compared to the control groups (K2–K3). Detailed statistics of the 5-HIAA/5-HT turnover ratios can be found in Supplementary Fig. 13.

## Discussion

Parkinson’s disease belongs to a group of neurodegenerative diseases that is directly related to the progressive loss of nigrostriatal dopaminergic neurons located in the SN [41]. The effect of the occurring neuropathological changes is a comprehensive disturbance of the balance between neurotransmitters of the extrapyramidal system associated with the occurrence of clinical motor symptoms. Therefore, motor impairments of Parkinson’s disease are often heralded by non-motor symptoms, which results in the clinical expression of the highly heterogeneous Parkinson syndrome. Progressive neurodegenerative changes are associated with changes in the functioning and regulation of the local systemic immune response, which seem to be one of the most prominent pathophysiological mechanisms accompanying Parkinson’s disease [42]. In this case, interconnected pathogenic neurodegenerative mechanisms are associated with the stimulation of microglial and astroglial cell populations, as well as with an increase in the expression level and synthesis of a number of inflammatory mediators such as cytokines, chemokines, acute phase proteins, adhesion molecules, complement system proteins, and growth factors [43]. The development of the neuroinflammatory reaction significantly contributes to the intensification and degeneration of dopaminergic neurons, consequently leading to the occurrence of comprehensive neurochemical disorders within the nigrostrial pathways. The actual trend regarding the research of Parkinson’s disease are based on experiments involving direct intracerebral administration using stereotactic methods of active forms of growth factors, neuromorphogens, cytokines, neuroprotective compounds, and recombinant forms of derivatives of these compounds to the deep neuroanatomical structures of the brain or to the ventricular system [44]. The essence of these directions of research and at the same time a challenge in the treatment of Parkinson’s disease is in this case the optimization and improvement of pharmacological treatment as well as the further development of regenerative medicine in the field of possibly permanent replenishment of DA deficiency by rebuilding neurons in the nigrostrial system, ultimately influencing the clinical improvement of treated patients [45].

Research studies published so far on preclinical models suggest that that the anti-inflammatory effect of Atsttrin in the pharmacodynamic sense is potentially superior to PGRN itself and other well-known TNFα antagonists [46, 47]. Ideally, treatment strategies of Parkinson’s disease should “stay ahead” of the neurodegenerative pathomechanisms through strategic application of different agents and that move beyond the traditional goal of symptomatic treatment to one of the causal treatment or disease progression control. Reflecting on this, we have performed the study in an attempt to elucidate the complex effect of direct intracerebral infusion of Atsttrin into ST using stereotactic methods in the preclinical C57BL/6 mouse model of Parkinson’s disease inducted by intraperitoneal MPTP intoxication. In this case, we have first attempted to validate the method of intracerebral stereotactic administration of Atsttrin in preclinical C57BL/6 mouse model settings. Furthermore, we have undertaken a detailed investigation aimed at estimation and optimization of effective and safety-related dose of Atsttrin delivered this route. Then, we have performed preliminary dose-dependent evaluation of parameters and markers regarding neurodegenerative process and development of inflammatory response. An additional derived aim of the study was to supplement the knowledge regarding the pharmacological mechanisms of action of Atsttrin and outline critical areas for future studies in the neuroscience field.

The panel of evaluated cytokines such as IL-1α, TNFα, and IL-6 included those which are characterized by the most prominent neuroinflammatory effects, which can also influence the scope of neurodamage indirectly by activating several intracellular signaling pathways including crucial NF-κB factor [48]. It is obvious that from the point of view of the study, Atsttrin is a molecule that directly antagonizes TNFα, which clearly directed the course of analysis towards a thorough evaluation of this signaling axis. When analyzing the expression profile of these cytokines, it should first be noted that intraperitoneal intoxication with MPTP in C57BL/6 mice was associated with an overall mean increase of IL-1α, TNF-α, and IL-6 mRNA expression levels within the majority of the evaluated neuroanatomical structures among the control groups. This observation is consistent with the previous published results in the literature describing the activation of neuroinflammatory mechanisms and remains a reference point for the purpose of the analysis, which was to identify adequate differences in the expression levels of these cytokines after intracerebral stereotaxic administration of Atsttrin [49]. Taking into account the results obtained during the analysis of the dose dependency effects of the increasing doses of Atsttrin, it was found that in the case of IL-1α mRNA expression level, the most effective dose was 0.5 μg (0.125 μg/μL), which caused a significant statistical decrease in the expression level of this cytokine within ST. Considering the obtained IL-1α mRNA expression profile, it should be clearly noted that the use of Atsttrin was associated with an obvious neuroprotective effect and control of the neuroinflammatory reaction. IL-1α is the primary cytokine associated with the development of the neuroinflammatory reaction and the pathogenesis of Parkinson’s disease [50]. Furthermore, post-mortem analyses showed that the expression level of IL-1α in the tissue obtained from SN is increased [51]. Results carried out on various preclinical models of neurodegeneration indicate a mutual correlation between the degree of neuronal death and expression level of IL-1α [52]. Moreover, the systemic and central administration of exogenous IL-1α to the structures of the nigrostrial system was also associated with the induction of neurodegenerative changes [53]. These changes could be consecutively inhibited or reversed when an IL-1α antagonist, such as IL-1 receptor antagonist (IL-1RA), is used [54]. At the same time, it was observed that IL-1α can influence neurodegenerative processes in the cerebral cortex via polysynaptic limbic pathways by intensifying glutamatergic (Glu) transmission [55]. The obtained results of anti-inflammatory effect of Atsttrin are consistent with the results obtained by Liu et al., who analyzed a model of direct intraventricular administration of LPS at a dose of 10 μg/μL in C57BL/6 mice [23]. In this case, Atsttrin at a dose of 2.5 mg/kg was administered intraperitoneally every 3 days for a period of 7 days before intraventricular administration of LPS. As part of this experiment, astrocyte cultures that were subjected to LPS at a dose of 0, 100, or 300 ng/mL and simultaneously Atsttrin at a dose of 200 ng/mL. In this case, the use of Atsttrin was associated with a reduction in the expression level of IL-1β and other inflammatory mediators in animals and within cell culture. Taking into account the results obtained during the analysis of the dose dependency effects of the increasing doses of Atsttrin, it was found that in the case of TNFα there were no observed statistically significant differences in the TNFα mRNA expression level within the ST after administration of all five doses of Atsttrin. Only one statistically significant difference included decrease of the TNFα mRNA expression level within the CA after intracerebral administration of Atsttrin into the ST at a dose of 0.1 μg (0.025 μg/μL). In this case, the observable trend seems to be important, whereas we have observed decrease of the level of TNFα mRNA expression after the administration of Atsttrin, which was the most prominent within the ST after administration of the two lowest doses 0.1 μg (0.025 μg/μL) and 0.5 μg (0.125 μg/μL). Considering the obtained TNFα mRNA expression profile, it should be noted that despite the lack of statistical significance the use of Atsttrin holds potentially neuroprotective effect that could control the neuroinflammatory reaction. The lack of statistical significance could be explained by a limitation in animals used, as well as observation time regarding the development of neuroinflammatory and neurodegenerative changes. An extremely important property of Atsttrin is its previously described ability to influence TNFα-related transmission and affinity to TNFR1 and TNFR2 exerting a direct anti-inflammatory effect. The previously described data and results of studies analyzing the role of TNFα clearly indicate the role of this cytokine in the pathogenesis of Parkinson’s disease, taking into account its increased level of expression in the nigrostrial system, cerebrospinal fluid (CSF) and peripheral blood observed both in humans and animals [56]. The expression level of TNFα is itself a factor that correlates with the severity of neurological symptoms in patients with Parkinson’s disease [57]. Regarding various murine models of MPTP intoxication, it was shown that knockout mice lacking the TNFα gene showed a lower degree of damage to the nigrostrial system [58]. Analogous results were obtained when using TNFα inhibitors, where a reduction in the degree of neurodegeneration was observed [59]. In the previously cited study by Liu et al., the expression level of TNFα was also analyzed accordingly [23]. Analogous to the level of IL-1α expression, the level of TNFα expression was reduced after using Atsttrin at a dose of 2.5 mg/kg when administered to C57BL/6 mice receiving LPS intraventricularly at a dose of 1 μL (10 μg/μL). In this case, it seems that the inhibition of the expression of TNFα family cytokines by Atsttrin is associated with a reduction in the degree of neuronal damage and activation of astro- and microglial cells. Moreover, our analyses show that Atsttrin at a dose of 0.5 μg (0.125 μg/μL) does not have neurotoxic effects or induces neurodegenerative phenomena related to the expression of TNFα and is potentially relatively safe in terms of its potential use in pharmacotherapy. Taking into account the results obtained during the analysis of the dose dependency effects of the increasing doses of Atsttrin, it was found that in the case of IL-6, there were no observed statistically significant differences in the IL-6 mRNA expression level within all the evaluated neuroanatomical regions after administration of all five doses of Atsttrin. In this case, the observable trend seems to be important, where we have observed a decrease in the level of IL-6 mRNA expression after the administration of Atsttrin, which was the most prominent after the administration of the highest doses. IL-6 constitutes one of the main cytokines with inflammatory and immunomodulatory effects; however, according to the available literature data, it may also inhibit the development of the neuroinflammatory reaction and thus potentially protect the nigrostriatal system [60]. Consistent with the previous data discussing the elevated levels of IL-6 in the CSF in patients with Parkinson’s disease, it can be concluded that our observations could be potentially related to the occurrence of a non-specific compensatory mechanism which promotes a neuroregenerative phenotype [61]. Additionally, in vitro cell culture studies of dopaminergic cells showed that the gradual addition of IL-6 in the concentration range of 5 to 50 ng/mL was associated with the prolonged survival of these cells [62]. What seems interesting in this case is the fact that knockout C57BL/6 mice lacking the IL-6 gene showed greater susceptibility to MPTP intoxication in terms of the development of the neuroinflammatory reaction and the synthesis of its associated mediators as well as an increased degree of DA loss [63]. The panel of evaluated enzymes associated with the metabolism of neurotransmitters and development neurodegenerative changes covered TH and TG2. In this case, TH is a key enzyme of DA metabolism, responsible for the conversion of L-tyrosine (L-Tyr) to L-3,4-dihydroxyphenylalanine (L-DOPA) and an important indicator of neurodegenerative phenomena [64]. In turn, TG2 is an enzyme involved in neurodegenerative processes, promoting the formation of high-molecular protein aggregates of alpha synuclein (ASN), which presence constitutes equivalent of the severity of neuropathological changes [65]. When analyzing the expression profile of these enzymes, it should first be noted that intraperitoneal intoxication with MPTP in C57BL/6 mice was associated with an overall mean decrease of TH and mean increase of TG2 mRNA expression levels within the majority of the evaluated neuroanatomical structures among the control groups. This observation is consistent with the previous published results in the literature describing the alternations in expression level of TH and TG2 and remains a reference point for the purpose of the analysis, which was to identify adequate differences in the expression levels of these enzymes after intracerebral stereotaxic administration of Atsttrin [66]. Taking into account the results obtained during the analysis of the dose dependency effects of the increasing doses of Atsttrin, it was found that in the case of TH mRNA expression level, the most effective dose was 1 μg (0.25 μg/μL), which caused a significant statistical increase in the expression level of this enzyme in within ST. These results would indicate the achievement of a very desirable and expected effect, which is the restoration of the DA synthesis function within the ST after the use of Atsttrin. This phenomenon may be related to the partial regeneration of dopaminergic cells as well as the activation of compensatory mechanisms in primary undamaged neurons that complement TH deficiencies [67]. Analogically, similar conclusions were observed, when C57BL/6 J mice were subjected to MPTP intoxication, followed by direct intraventricular administration of 1 ng PGRN [68]. In this case, an increase in TH expression was found in the immunochemical analyses of brains collected from animals on the third day after the experimental procedures. Taking into account the results obtained during the analysis of the dose dependency effects of the increasing doses of Atsttrin, it was found that in the case of TG2 mRNA expression level, there were no observed significant statistical differences regarding expression level of this enzyme within ST, CX, and CM, apart from the statistically significant increase within the CA after administration of the 0.5 μg (0.125 μg/μL) dose. TG2 possesses several cell biological functions and enzymatic activities, its trans-amidation activity, which is responsible for the cross-linking of proteins between glutamine (Gln) and lysine (Lys), producing inter- or intramolecular covalent bonds which prevent proteolysis of ASN [69]. The TG2-mediated cross-linking is responsible for the creation of a more toxic form of ASN. The increased presence of TG2 protein and mRNA expression was observed within SN and CSF in patients with Parkinson’s disease [70]. Based on the obtained results, it is difficult to clearly determine how TG2 expression is related to the signaling pathways activated via PGRN/Atsttrin. This observation draws attention to the need to further investigate the role of the potential interaction of TG2 and PGRN in the context of cells of the nigrostrial system. The evaluated neurochemical profile included concentrations of DA, DOPAC, 3-MT, HVA, NA, MHPG, 5-HT, and 5-HIAA as well as the metabolic turnover regarding DOPAC/DA, 3-MT/DA, 3-MT/DA, HVA/DA, MHPG/NA, and 5-HIAA/5-HT ratios. A comprehensive evaluation of the changes in the central neurochemical profile during the study was performed in order to initially assess the functions and interactions of the dopaminergic, noradrenergic, and serotonergic systems. The level of DA concentration as the main neurotransmitter involved in the pathophysiology of Parkinson’s disease is in this case the most prominent and useful indicator of the extent of neurodamage [71]. From the point of view of the hypothesis of the study, Atsttrin was considered an agent which possesses the protective and regenerative potentials of dopaminergic neurons, which clearly directed the course of the analyses. 

When analyzing the concentration profile of DA, it should first be noted that intraperitoneal intoxication with MPTP in C57BL/6 mice was associated with an overall mean decrease of DA concentration level within all majority of the evaluated neuroanatomical structures among the control groups. This observation is consistent with the previous published results in the literature describing the significant DA depletion and supporting the apoptotic theory in Parkinson’s disease remaining as a reference point for the purpose of the analysis, which was to identify adequate differences in the concentration levels of this neurotransmitter after intracerebral stereotaxic administration of Atsttrin [72]. Taking into account the results obtained during the analysis of the dose dependency effects of the increasing doses of Atsttrin, it was found that in the case of DA, the use of doses of 0.5 μg (0.125 μg/μL) and 1 μg (0.25 μg/μL) was paradoxical to the expected effect, associated with a statistically significant decrease of this neurotransmitter within ST. Analogous observations regarding the concentration level of DA in other neuroanatomical structures did not show clear statistical significance. In this case, the observable trend seems to be important, where we have observed an increase of DA concentration level after the administration of Atsttrin, which was the most prominent after the administration of the highest doses.

Taking into account the results obtained during the analysis of the dose dependency effects of the increasing doses of Atsttrin, it was found that in the case of DOPAC/DA, 3-MT/DA, and HVA/DA turnover ratios, the use of doses of 0.5 μg (0.125 μg/μL), 1 μg (0.25 μg/μL) and 2 μg (0.5 μg/μL) was associated with a statistically significant increase of these turnover ratios within ST. Analogous observations regarding the DOPAC/DA, 3-MT/DA, and HVA/DA turnover ratios in other neuroanatomical structures did not show clear statistical significance. Based on the obtained results, a preliminary paradoxical conclusion can be drawn, which shows that the use of Atsttrin does not show a neuroprotective effect in the analyzed C57BL/6 mice model of Parkinson’s disease. In this case, we observed unexpected incoherence in the obtained results regarding mRNA expression of immune system mediators and monoamine concentration levels. In the study conducted on analogous animal model of Parkinson’s disease by van Kampen et al., the therapeutic intervention of the experiment covered direct stereotaxic administration of a viral vector containing the PGRN gene [73]. The vector used during the experiment was pLenti6/R4R2/V5-DEST (ND-602) containing the PGRN gene, while C57BL/6 mice were subjected to intraperitoneal intoxication with MPTP at a dose of 30 mg/kg for 5 consecutive days. In this case, the direct intracerebral injection of the ND-602 lentiviral vector was associated with no significant decrease in the levels of DA, DOPAC, and HVA concentrations and no increase in the levels of DOPAC/DA and HVA/DA turnover ratios in within ST. At the same time, the administration of ND-602 was associated with the lack of a significant decrease in the population of TH + cells within the ST and SN, collectively demonstrating that PGRN has a neuroprotective effect on cells of the nigrostrial system. The literature data has shown that TNFα can exert its neuroprotective effect through TNFR1 receptor, in this case modulating the dopamine transporter (DAT) function, which was observed in animal model of Parkinson’s disease induced through methamphetamine administration [74]. It is known that receptors dedicated to TNFα are located on the surface of all cellular elements occurring within the nigrostrial system, which is related to the fact that this cytokine can affect dopaminergic neurons directly and indirectly by affecting glial cells [75]. The increase in the expression of the TNFR1 receptor pool within the nigrostrial system of in patients with Parkinson’s disease was observed [76]. Therefore, the expected effect of Atsttrin administration should cover the reduction of the effect of TNFα at the initial stage after MPTP intoxication and should be associated with a significant, lasting reduction in the degree of neurodegeneration. However, it cannot be ruled out that in this case TNFα secreted in the acute phase of the neuroinflammatory reaction may play a role in activating repair processes [77]. At the same time, the synthesis of this cytokine may be associated with the intensification of changes within the damaged nigrostrial pathway, unfavorable for dopaminergic neurons. The early use of Atsttrin and the associated lack of the expected pharmacological effect can be associated with the pathophysiological mechanisms described above. Additionally, the action of TNFα is also mediated by other receptors, where blocking TNFR1 may force compensatory mechanisms involving the synthesis of other functional receptors, thus maintaining the TNFα-related signal, leading to a reduction in DA concentration level [78]. Some explanation for the obtained results may be provided by observations regarding the functioning of TNFα within the CNS, where its varied impact is observed depending on the given neuroanatomical location and the ability to activate transcription factors such as NF-κB. At the same time, it is known that TNFα can regulate the level of DA concentration within synapses and nerve cell bodies; therefore, it is worth noting that the use of the HPLC method to analyze the concentration of neurotransmitters in homogenized tissue covered the value of the total pool of DA present in the cytoplasm and extracellular space and stored in synaptic vesicles, which may influence the actual postulated mechanism of Atsttrin pharmacological action under the conditions of the discussed model [79]. It cannot also be ruled out that extending the observation time could potentially show a subsequent increase in DA concentration, which, taking into account the latency, could be sufficient to fully activate neuroregenerative mechanisms.

The degeneration of the noradrenergic system in the course of Parkinson’s disease is associated with a broad spectrum encompassing autonomic, behavioral, and cognitive parameters [80]. Therefore, the noradrenergic dysfunction in Parkinson’s disease was associated with the promotion of inflammatory response, diminishing neurotrophic factors, and excessive oxidation within SN [81]. Accordingly, the complex evaluation of NA and MHPH concentration as well as its turnover ratio is reasonable regarding complex interplay between dopaminergic and noradrenergic systems.

When analyzing the concentration profile of NA and MHPG, it should first be noted that intraperitoneal intoxication with MPTP in C57BL/6 mice was associated with an overall mean decrease of NA and mean increase of MHPG concentration level within all majority of the evaluated neuroanatomical structures among the control groups. This observation is consistent with the previous published results in the literature describing the significant NA depletion remaining as reference point for the purpose of the analysis, which was to identify adequate differences in the concentration levels of this neurotransmitters after intracerebral stereotaxic administration of Atsttrin [82]. Taking into account the results obtained during the analysis of the dose dependency effects of the increasing doses of Atsttrin, it was found that in the case of NA, the use of dose of 0.1 μg/4 μL (0.025 μg/μL) was associated with a statistically significant decrease of this neurotransmitter within CA and CX. The use of doses of 0.1 μg/4 μL (0.025 μg/μL) and 0.5 μg/4 μL (0.125 μg/μL) was associated with a statistically significant increase of NA within CM. Therefore, the use of doses of 1 μg/4 μL (0.25 μg/μL), 2 μg/4 μL (0.5 μg/μL), and 5 μg/4 μL (1.25 μg/μL) was associated with a statistically significant decrease of NA within CM. The use of doses of 1 μg/4 μL (0.25 μg/μL) and 2 μg/4 μL (0.5 μg/μL) was associated with a statistically significant decrease of MHPG/NA turnover ratio. In the natural course of Parkinson’s disease, apart from the neuropathological changes in the dopaminergic pathways, degeneration of pigment cells is also observed in the area of the locus coeruleus located in the pons, where clusters of noradrenergic cells are located [83]. Furthermore, it is known, that damage to the locus coeruleus area is associated with inhibition of DA synthesis within the ST, while stimulation of this neuroanatomical structure increases the activity of dopaminergic neurons [84]. Taking into account the pathophysiological grounds assumed in this study, it would seem that the use of Atsttrin should be associated with limiting the decrease in the level of NA concentration after MPTP intoxication within neuroanatomical structures related to noradrenergic transmission. Based on the current research results, it is difficult to clearly determine the mechanism by which Atsttrin affects noradrenergic transmission in the analyzed model. It seems reasonable that in this case analogous pathophysiological phenomena responsible for the previously observed decrease in DA levels after the use of Atsttrin should be partially taken into account regarding observed changes of NA concentrations levels.

Serotonergic neurotransmission is widely distributed within the CNS and mediated by neurons of the raphe nuclei [85]. Besides motor complications, serotonergic pathology has been hypothesized as a crucial mechanism underlying non-motor symptoms in Parkinson’s disease [86]. Consequently, accordingly, the complex evaluation of 5-HT and 5-HIAA concentration as well as its turnover ratio is reasonable regarding the complex interplay between dopaminergic and serotonergic systems.

When analyzing the concentration profile of 5-HT and 5-HIAA, it should first be noted that the intraperitoneal intoxication with MPTP in C57BL/6 mice was associated with an overall mean increase of this monoamines concentration level within all majority of the evaluated neuroanatomical structures among the control groups. This observation is consistent with the previous published results in the literature describing the relative resistance of serotonergic neurons to MPTP intoxication remaining as a reference point for the purpose of the analysis, which was to identify adequate differences in the concentration levels of this neurotransmitters after intracerebral stereotaxic administration of Atsttrin [87]. Taking into account the results obtained during the analysis of the dose dependency effects of the increasing doses of Atsttrin, it was found that in the case of 5-HT, the use of dose of 0.1 μg/4 μL (0.025 μg/μL), 1 μg/4 μL (0.25 μg/μL), and 2 μg/4 μL (0.5 μg/μL) was associated with a statistically significant decrease of this neurotransmitter within CM. In the course of Parkinson’s disease, a decrease in 5-HT levels is observed, which is usually most expressed in the ventral tegmental area and other mesolimbic structures [88]. In this case, patients with severe apathy, depression, and anxiety, i.e., showing comprehensive neurocognitive disorders in the course of Parkinson’s disease, exhibit impaired serotonergic transmission within the insula, orbital cortex, and anterior cingulate gyrus, especially in the area located ventral to the genu of corpus callosum [89]. The decrease in DA activity in the course of Parkinson’s disease also results in a decrease in the activity of serotonergic transmission [90]. On this terms, antagonism towards the 5-HT_2C_ receptor seems to be important, which is associated with the intensification of DA secretion in the brain prefrontal area [91]. This receptor plays an important role in regulating DA release, both directly and through glutamatergic projections to the nucleus accumbens [92]. The effect of 5-HT in this case seems to be responsible for the inhibition of Glu through the 5-HT_1A_ receptor [93]. In this case, it seems that Atsttrin has a positive effect on the restoration of serotonergic transmission in the analyzed neuroanatomical areas undergoing the neurodegeneration process in the course of Parkinson’s disease. In recent years, the increasing number of studies has shown that Atsttrin exerts protective and anti-inflammatory effects on various evaluated disease models [94]. Future studies should assess the pharmacological potential of Atsttrin to simultaneously address the multidimensional aspects of neurodegenerative diseases. Nonetheless, our findings provided strong evidence that Atsttrin may have therapeutic potential for the treatment of Parkinson’s disease. The rational selection and validation of the appropriate dose of Atsttrin, taking into account a wide panel of determined parameters as therapy targets, was a decision dictated by a compromise between the potential observed neuroprotective and neurotoxic effects of successively increasing doses of the compound. According to the results data obtained from the analysis of the overall impact of the increasing doses of Atsttrin on the dose–response and dose–effect relationships, a dose of 0.5 μg (0.125 μg/μL) of the compound was selected. The phenomena described after the use of 0.5 μg (0.125 μg/μL) dose of Atsttrin in experimental model of Parkinson’s disease induced by MPTP in C57BL/6 mice could in this case subjectively reflect the anti-inflammatory effects demonstrated in previous studies in other models of various disease entities. Collectively, Atsttrin could be considered a therapeutic agent in the therapy of Parkinson’s disease via targeting TNFα and in this case could be a future novel drug candidate for the treatment of neurodegenerative diseases.

## Supplementary Information

Below is the link to the electronic supplementary material.

Supplementary file1 (DOCX 541 KB)

## Data Availability

No datasets were generated or analyzed during the current study.
