# Supplementary material for: Dose-Ranging Effects of the Intracerebral Administration of Atsttrin in Experimental Model of Parkinson’s Disease Induced by 1-Methyl-4-phenyl-1,2,3,6-tetrahydropyridine (MPTP) in Mice
Source: Mol Neurobiol. 2024 Apr 20;61(11):9432–58. doi: 10.1007/s12035-024-04161-0 (PMC11496375; doi:10.1007/s12035-024-04161-0)
Supplement: Supplementary file 1 — Supplementary file1 (DOCX 541 KB) [file 12035_2024_4161_MOESM1_ESM.docx]

**ORIGINAL ARTICLE**

**Dose-ranging effects of the intracerebral administration of Atsttrin in experimental model of Parkinson's disease induced by 1-methyl-4-phenyl-1,2,3,6-tetrahydropyridine (MPTP) in mice**

**Łukasz A. Poniatowski^1,2^ · Ilona Joniec-Maciejak^1^ · Adriana Wawer^1^ · Anna Sznejder-Pachołek^1^ · Ewa Machaj^1^ · Katarzyna Ziętal^1^ · Dagmara Mirowska-Guzel^1^**

**^1^**Department of Experimental and Clinical Pharmacology, Centre for Preclinical Research and Technology (CePT), Medical University of Warsaw, Banacha 1B, 02-097 Warsaw, Poland

**^2^**Department of Neurosurgery, Dietrich-Bonhoeffer-Klinikum, Salvador-Allende-Straße 30, 17036 Neubrandenburg, Germany

**Corresponding author:**

Łukasz A. Poniatowski, e-mail: lukasz.poniatowski@gmail.com

Department of Experimental and Clinical Pharmacology, Centre for Preclinical Research and Technology (CePT), Medical University of Warsaw, Banacha 1B, 02-097 Warsaw, Poland

Phone: +48 22 116-61-60 **|** Fax: +48 22 116-62-02

www.wum.edu.pl **|** www.wum.edu.pl/en/english **|** www.cept.wum.edu.pl

Department of Neurosurgery, Dietrich-Bonhoeffer-Klinikum, Salvador-Allende-Straße 30, 17036 Neubrandenburg, Germany

Phone: +49 395 775-2891**|** Fax: +49 395 775-2889

www.dbknb.de **|** www.dbknb.de/nch

**Co-authors:**

Ilona Joniec-Maciejak, e-mail: ijoniec@wum.edu.pl

Adriana Wawer, e-mail: adriana.wawer@wum.edu.pl

Anna Sznejder-Pachołek, e-mail: annas.pacholek@gmail.com

Ewa Machaj, e-mail: ewa.machaj@wum.edu.pl

Katarzyna Ziętal, e-mail: katarzyna.zietal@wum.edu.pl

Dagmara Mirowska-Guzel, e-mail: dmirowska@wum.edu.pl

Supplementary Figure 1. Changes in the concentration of DA assessed after intracerebral administration into ST of five increasing doses of Atsttrin using stereotactic methods in C57BL/6 mice subjected to MPTP intoxication within ST (A), CA (B), CX (C) and CM (D). The sample concentration was expressed as pg/mg wet tissue. The results were presented as mean values ± SEM. * - difference from the appropriate control group (K2), *p<0.05, **p<0.01, ***p<0.001; # - difference from the appropriate control group (K3), ^#^p<0.05; ^##^p<0.01; ^###^p<0.001

**K1**

**K2**

**K3**

**P1**

**P2**

**P3**

**P4**

**P5**

**K1**

**K2**

**K3**

**P1**

**P2**

**P3**

**P4**

**P5**

**K1**

**K2**

**K3**

**P1**

**P2**

**P3**

**P4**

**P5**

**K1**

**K2**

**K3**

**P1**

**P2**

**P3**

**P4**

**P5**

**0**

**0**

**0**

*****

**Control group not subjected to any intervention (7d)**

**4x MPTP-HCL (7d)**

**Ringer’s solution (4 μL) → ST + 4x MPTP-HCL (7d)**

**Atsttrin 0.1 μg/4 μL (0.025 μg/μL) → ST + 4x MPTP-HCL (7d)**

**DA concentration – ST**

**0**

**Atsttrin 0.5 μg/4 μL (0.125 μg/μL) → ST + 4x MPTP-HCL (7d)**

**Atsttrin 1 μg/4 μL (0.25 μg/μL) → ST + 4x MPTP-HCL (7d)**

**Atsttrin 5 μg/4 μL (1.25 μg/μL) → ST + 4x MPTP-HCL (7d)**

**x**

**TP**

**(**

**)**

**L (7d)**

**Kontrola niepoddana żadnej procedurze i int**

**rwencji (7d)**

*****

*****

**D)**

**B)**

**A)**

**Atsttrin 2 μg/4 μL (0.5 μg/μL) → ST + 4x MPTP-HCL (7d)**

**(0.5 μg**

**μ**

**)**

**4**

**P**

**C**

**L (7d)**

**Kontrola niepoddana żadnej procedurze i int**

**rwencji (7d)**

*****

*****

**D)**

**B)**

**A)**

**DA concentration – CA**

**DA concentration – CX**

**DA concentration – CM**

**DA concentration [pg/mg]**

**DA concentration [pg/mg]**

**DA concentration [pg/mg x10^2^]**

**DA concentration [pg/mg]**

**80**


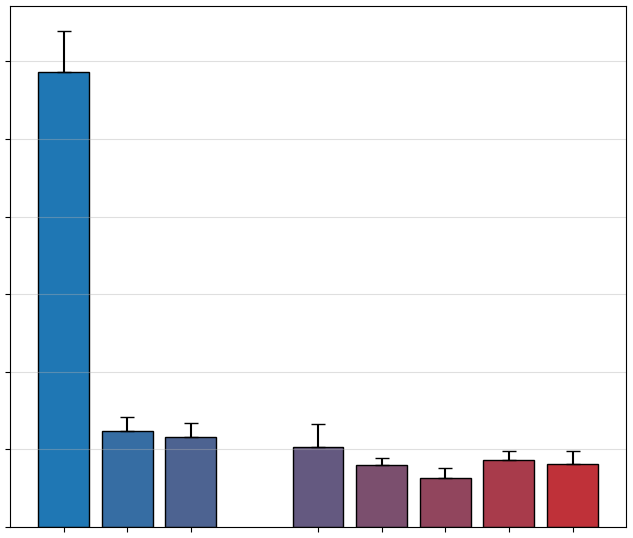


*****

*****

**#**

**100**

**60**

**40**

**20**


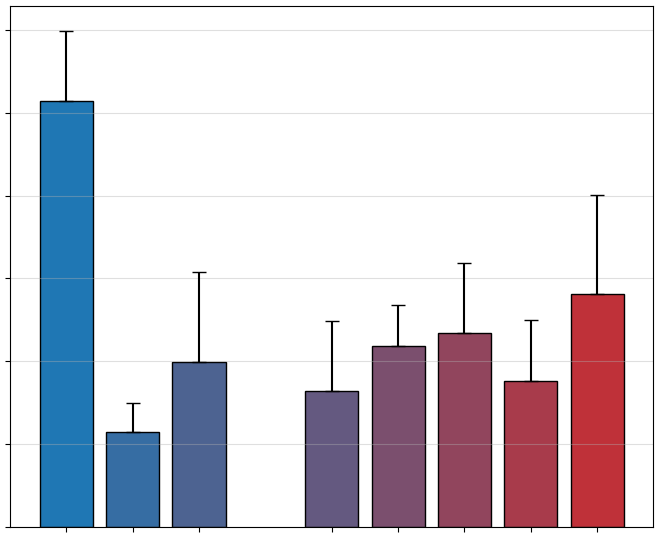


**A)**

**10**

**20**

**30**

**40**

**50**

**60**

**B)**


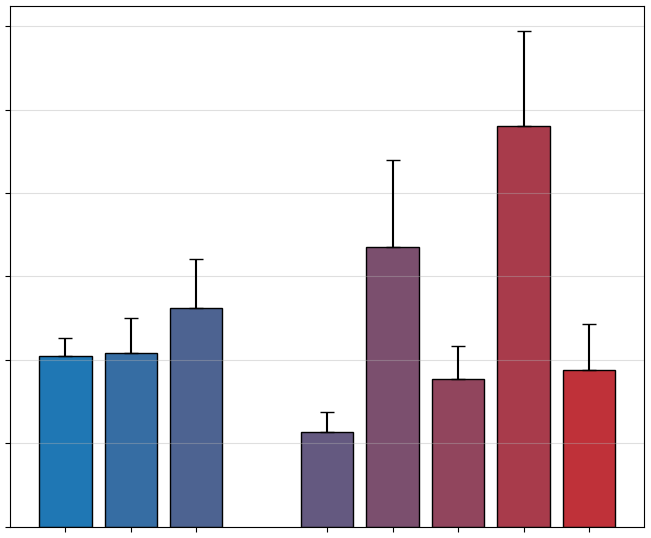


**80**

**120**

**100**

**60**

**40**

**20**

**C)**


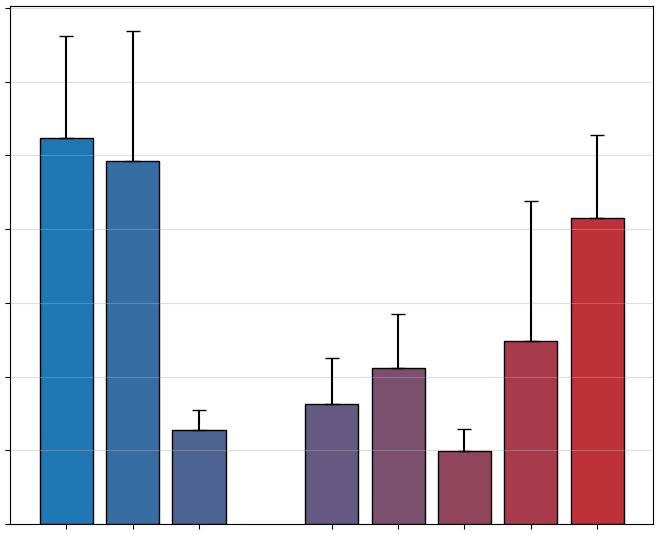


**4**

**6**

**10**

**14**

**12**

**8**

**2**

**D)**

**120**

Supplementary Figure 2. Changes in the concentration of DOPAC assessed after intracerebral administration into ST of five increasing doses of Atsttrin using stereotactic methods in C57BL/6 mice subjected to MPTP intoxication within ST (A), CA (B), CX (C) and CM (D). The sample concentration was expressed as pg/mg wet tissue. The results were presented as mean values ± SEM. * - difference from the appropriate control group (K2), *p<0.05, **p<0.01, ***p<0.001; # - difference from the appropriate control group (K3), ^#^p<0.05; ^##^p<0.01; ^###^p<0.001

**K1**

**K2**

**K3**

**P1**

**P2**

**P3**

**P4**

**P5**

**K1**

**K2**

**K3**

**P1**

**P2**

**P3**

**P4**

**P5**

**K1**

**K2**

**K3**

**P1**

**P2**

**P3**

**P4**

**P5**

**K1**

**K2**

**K3**

**P1**

**P2**

**P3**

**P4**

**P5**

**0**

**0**

**0**

*****

**Control group not subjected to any intervention (7d)**

**4x MPTP-HCL (7d)**

**Ringer’s solution (4 μL) → ST + 4x MPTP-HCL (7d)**

**Atsttrin 0.1 μg/4 μL (0.025 μg/μL) → ST + 4x MPTP-HCL (7d)**

**DOPAC concentration – ST**

**0**

**Atsttrin 0.5 μg/4 μL (0.125 μg/μL) → ST + 4x MPTP-HCL (7d)**

**Atsttrin 1 μg/4 μL (0.25 μg/μL) → ST + 4x MPTP-HCL (7d)**

**Atsttrin 5 μg/4 μL (1.25 μg/μL) → ST + 4x MPTP-HCL (7d)**

**x**

**TP**

**(**

**)**

**L (7d)**

**Kontrola niepoddana żadnej procedurze i int**

**rwencji (7d)**

*****

*****

**D)**

**B)**

**A)**

**Atsttrin 2 μg/4 μL (0.5 μg/μL) → ST + 4x MPTP-HCL (7d)**

**(0.5 μg**

**μ**

**)**

**4**

**P**

**C**

**L (7d)**

**Kontrola niepoddana żadnej procedurze i int**

**rwencji (7d)**

*****

*****

**D)**

**B)**

**A)**

**DOPAC concentration – CA**

**DOPAC concentration – CX**

**DOPAC concentration – CM**

**DOPAC concentration [pg/mg]**

**DOPAC concentration [pg/mg]**

**DOPAC concentration [pg/mg]**

**8**

**12**

*****

*****

**#**

**6**

**4**

**2**

**60**

**6**

**8**

**12**

**16**

**14**

**4**


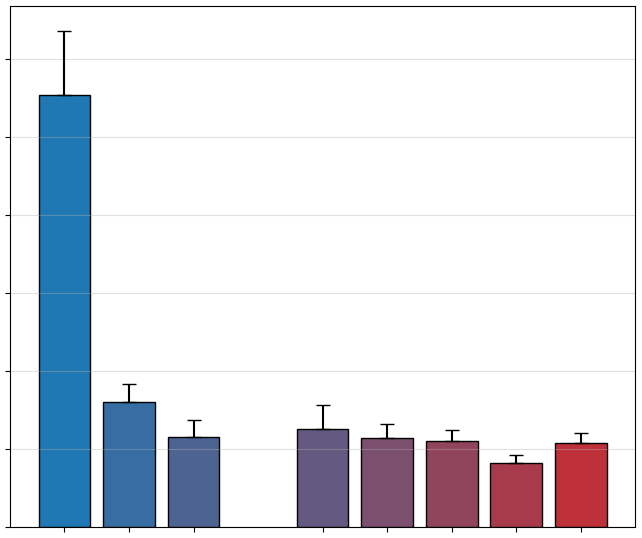


**DOPAC concentration [pg/mg x10^2^]**

**10**

**A)**


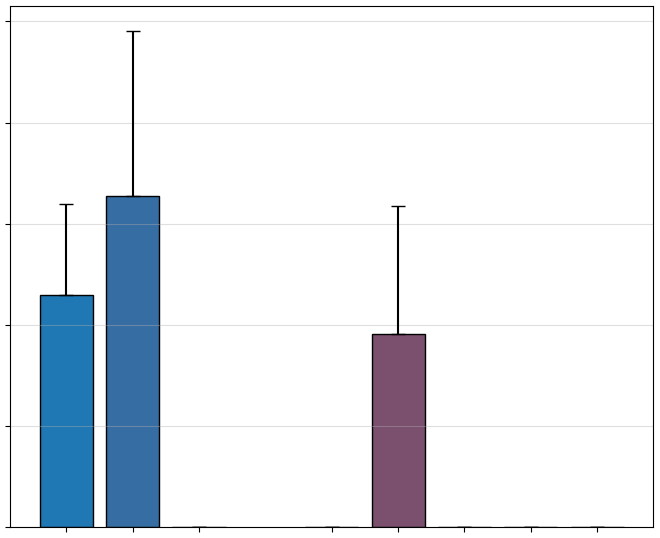


**8**

**6**

**4**

**2**

**10**

**50**

**40**

**30**

**20**

**10**


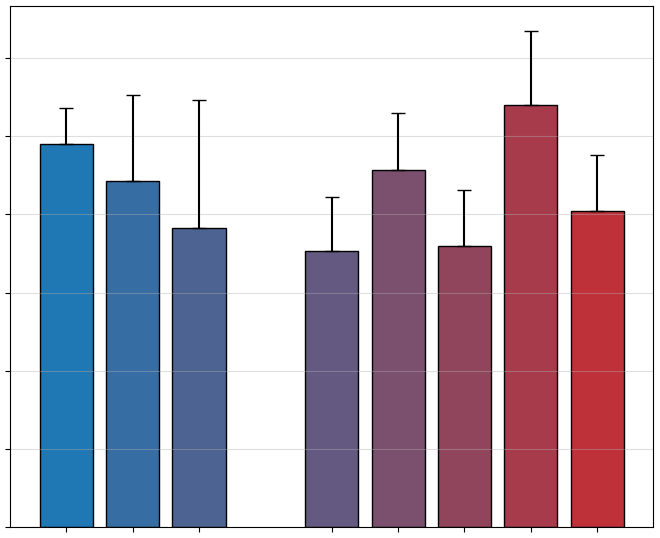


**B)**

**C)**


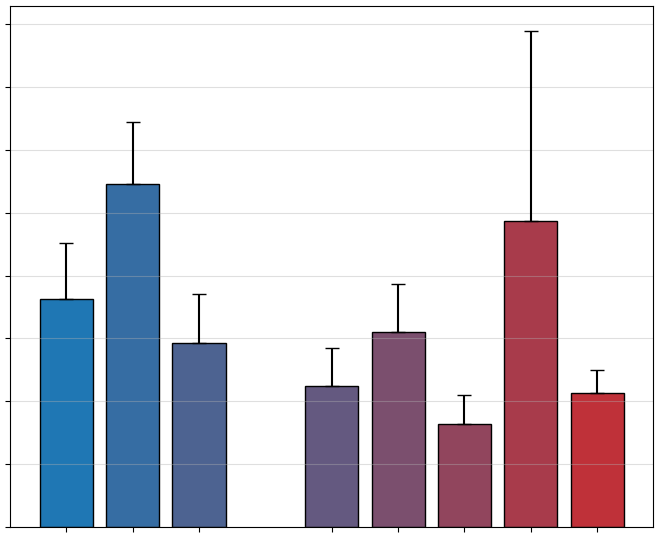


**10**

**2**

**D)**

*****

*****

******

*****

**K1**

**K2**

**K3**

**P1**

**P2**

**P3**

**P4**

**P5**

**K1**

**K2**

**K3**

**P1**

**P2**

**P3**

**P4**

**P5**

**K1**

**K2**

**K3**

**P1**

**P2**

**P3**

**P4**

**P5**

**K1**

**K2**

**K3**

**P1**

**P2**

**P3**

**P4**

**P5**

**0**

*****

**Control group not subjected to any intervention (7d)**

**4x MPTP-HCL (7d)**

**Ringer’s solution (4 μL) → ST + 4x MPTP-HCL (7d)**

**Atsttrin 0.1 μg/4 μL (0.025 μg/μL) → ST + 4x MPTP-HCL (7d)**

**3-MT concentration – ST**

**0**

**Atsttrin 0.5 μg/4 μL (0.125 μg/μL) → ST + 4x MPTP-HCL (7d)**

**Atsttrin 1 μg/4 μL (0.25 μg/μL) → ST + 4x MPTP-HCL (7d)**

**Atsttrin 5 μg/4 μL (1.25 μg/μL) → ST + 4x MPTP-HCL (7d)**

**x**

**TP**

**(**

**)**

**L (7d)**

**Kontrola niepoddana żadnej procedurze i int**

**rwencji (7d)**

*****

*****

**D)**

**B)**

**A)**

**Atsttrin 2 μg/4 μL (0.5 μg/μL) → ST + 4x MPTP-HCL (7d)**

**(0.5 μg**

**μ**

**)**

**4**

**P**

**C**

**L (7d)**

**Kontrola niepoddana żadnej procedurze i int**

**rwencji (7d)**

*****

*****

**D)**

**B)**

**A)**

**3-MT concentration – CA**

**3-MT concentration – CX**

**3-MT concentration – CM**

**3-MT concentration [pg/mg]**

**3-MT concentration [pg/mg]**

**3-MT concentration [pg/mg]**

**800**

*****

*****

**#**

**70**

**3-MT concentration [pg/mg]**

**5**

**25**

**60**

**50**

**40**

**30**

**20**

*****


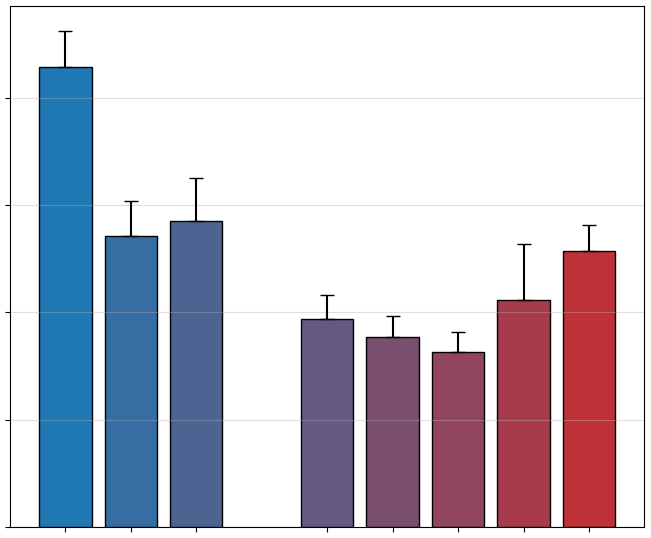


**600**

**400**

**200**

**A)**


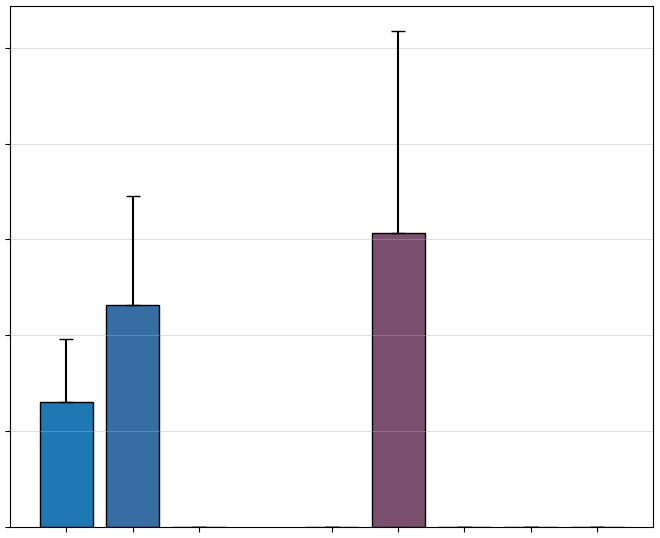


**0**

**20**

**15**

**10**


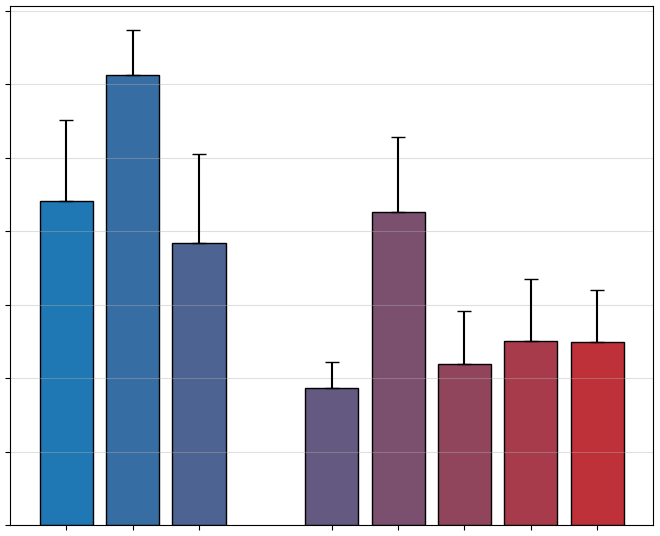


**10**

**B)**

**C)**


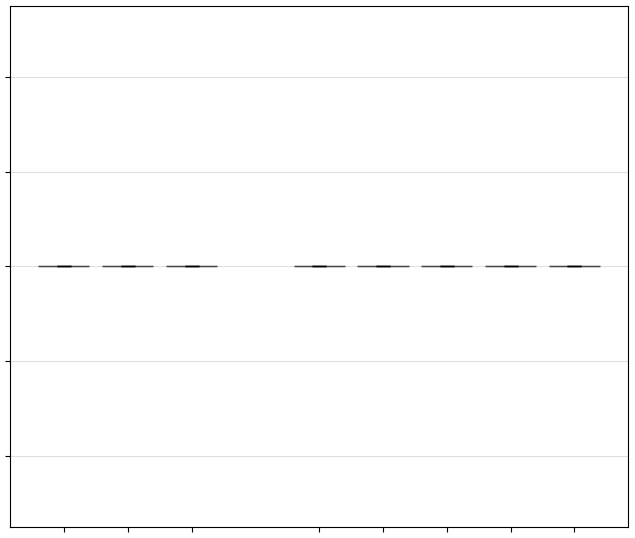


**-0.04**

**-0.02**

**0**

**0.02**

**0.04**

**D)**

*****

*****

**#**

*******

******

******

******

Supplementary Figure 3. Changes in the concentration of 3-MT assessed after intracerebral administration into ST of five increasing doses of Atsttrin using stereotactic methods in C57BL/6 mice subjected to MPTP intoxication within ST (A), CA (B), CX (C) and CM (D). The sample concentration was expressed as pg/mg wet tissue. The results were presented as mean values ± SEM. * - difference from the appropriate control group (K2), *p<0.05, **p<0.01, ***p<0.001; # - difference from the appropriate control group (K3), ^#^p<0.05; ^##^p<0.01; ^###^p<0.001

Supplementary Figure 4. Changes in the concentration of HVA assessed after intracerebral administration into ST of five increasing doses of Atsttrin using stereotactic methods in C57BL/6 mice subjected to MPTP intoxication within ST (A), CA (B), CX (C) and CM (D). The sample concentration was expressed as pg/mg wet tissue. The results were presented as mean values ± SEM. * - difference from the appropriate control group (K2), *p<0.05, **p<0.01, ***p<0.001; # - difference from the appropriate control group (K3), ^#^p<0.05; ^##^p<0.01; ^###^p<0.001

**K1**

**K2**

**K3**

**P1**

**P2**

**P3**

**P4**

**P5**

**K1**

**K2**

**K3**

**P1**

**P2**

**P3**

**P4**

**P5**

**K1**

**K2**

**K3**

**P1**

**P2**

**P3**

**P4**

**P5**

**K1**

**K2**

**K3**

**P1**

**P2**

**P3**

**P4**

**P5**

**0**

**Control group not subjected to any intervention (7d)**

**4x MPTP-HCL (7d)**

**Ringer’s solution (4 μL) → ST + 4x MPTP-HCL (7d)**

**Atsttrin 0.1 μg/4 μL (0.025 μg/μL) → ST + 4x MPTP-HCL (7d)**

**HVA concentration – ST**

**0**

**Atsttrin 0.5 μg/4 μL (0.125 μg/μL) → ST + 4x MPTP-HCL (7d)**

**Atsttrin 1 μg/4 μL (0.25 μg/μL) → ST + 4x MPTP-HCL (7d)**

**Atsttrin 5 μg/4 μL (1.25 μg/μL) → ST + 4x MPTP-HCL (7d)**

**x**

**TP**

**(**

**)**

**L (7d)**

**Kontrola niepoddana żadnej procedurze i int**

**rwencji (7d)**

*****

*****

**D)**

**B)**

**A)**

**Atsttrin 2 μg/4 μL (0.5 μg/μL) → ST + 4x MPTP-HCL (7d)**

**(0.5 μg**

**μ**

**)**

**4**

**P**

**C**

**L (7d)**

**Kontrola niepoddana żadnej procedurze i int**

**rwencji (7d)**

*****

*****

**D)**

**B)**

**A)**

**HVA concentration – CA**

**HVA concentration – CX**

**HVA concentration – CM**

**HVA concentration [pg/mg]**

**HVA concentration [pg/mg]**

**HVA concentration [pg/mg]**

**HVA concentration [pg/mg x 10^2^]**

**70**

**75**

**0**

**60**

**50**

**30**

**25**


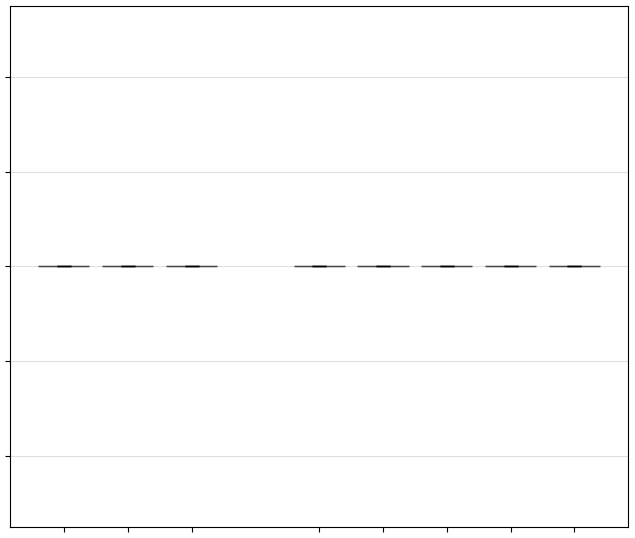


**-0.04**

**-0.02**

**0**

**0.02**

**0.04**

**D)**


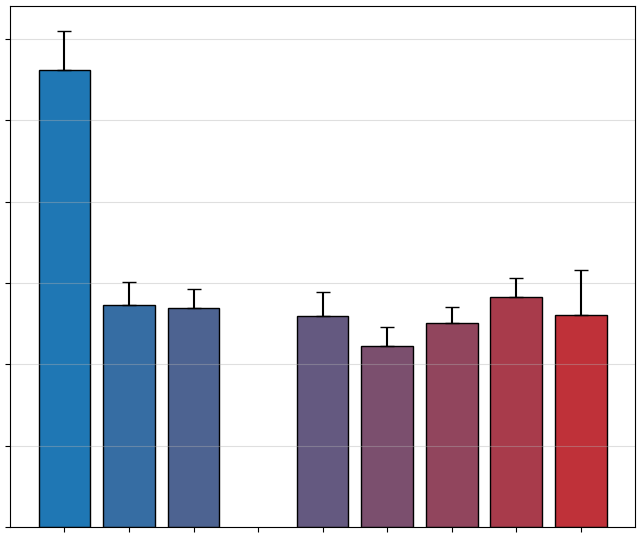


**A)**

**8**

**6**

**4**

**2**

**10**

**12**


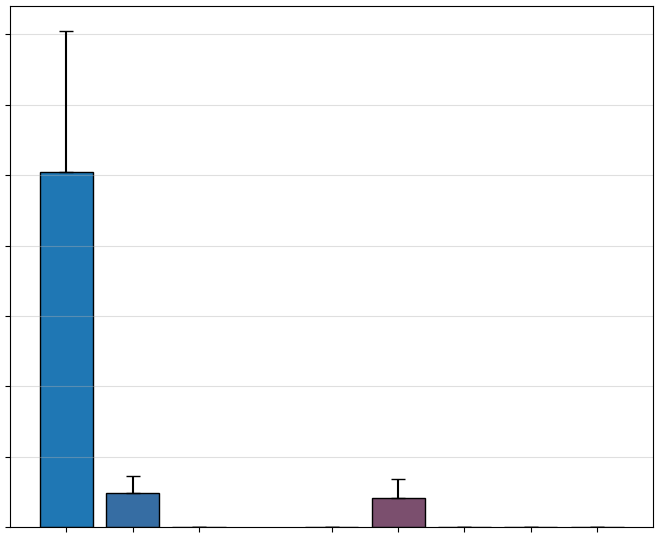


**40**

**20**

**10**

**B)**


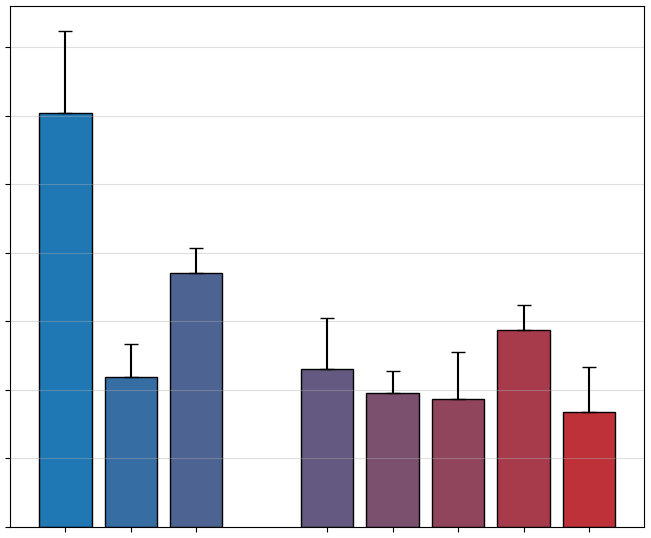


**175**

**150**

**125**

**100**

**50**

**C)**

**##**

**#**

**#**

Supplementary Figure 5. Changes in the turnover level of DOPAC/DA assessed after intracerebral administration into ST of five increasing doses of Atsttrin using stereotactic methods in C57BL/6 mice subjected to MPTP intoxication within ST (A), CA (B), CX (C) and CM (D). The results were expressed as the value of the concentration ratio of the analyzed monoamines. The results were presented as mean values ± SEM. * - difference from the appropriate control group (K2), *p<0.05, **p<0.01, ***p<0.001; # - difference from the appropriate control group (K3), ^#^p<0.05; ^##^p<0.01; ^###^p<0.001

**K1**

**K2**

**K3**

**P1**

**P2**

**P3**

**P4**

**P5**

**K1**

**K2**

**K3**

**P1**

**P2**

**P3**

**P4**

**P5**

**K1**

**K2**

**K3**

**P1**

**P2**

**P3**

**P4**

**P5**

**K1**

**K2**

**K3**

**P1**

**P2**

**P3**

**P4**

**P5**

**0**

**Control group not subjected to any intervention (7d)**

**4x MPTP-HCL (7d)**

**Ringer’s solution (4 μL) → ST + 4x MPTP-HCL (7d)**

**Atsttrin 0.1 μg/4 μL (0.025 μg/μL) → ST + 4x MPTP-HCL (7d)**

**DOPAC/DA turnover – ST**

**0**

**Atsttrin 0.5 μg/4 μL (0.125 μg/μL) → ST + 4x MPTP-HCL (7d)**

**Atsttrin 1 μg/4 μL (0.25 μg/μL) → ST + 4x MPTP-HCL (7d)**

**Atsttrin 5 μg/4 μL (1.25 μg/μL) → ST + 4x MPTP-HCL (7d)**

**x**

**TP**

**(**

**)**

**L (7d)**

**Kontrola niepoddana żadnej procedurze i int**

**rwencji (7d)**

*****

*****

**D)**

**B)**

**A)**

**Atsttrin 2 μg/4 μL (0.5 μg/μL) → ST + 4x MPTP-HCL (7d)**

**(0.5 μg**

**μ**

**)**

**4**

**P**

**C**

**L (7d)**

**Kontrola niepoddana żadnej procedurze i int**

**rwencji (7d)**

*****

*****

**D)**

**B)**

**A)**

**DOPAC/DA turnover – CA**

**DOPAC/DA turnover – CX**

**DOPAC/DA turnover – CM**

**DOPAC/DA ratio**

**DOPAC/DA ratio**

**DOPAC/DA ratio**

**DOPAC/DA ratio x 10^-2^**

**0**

**##**

**#**

**#**


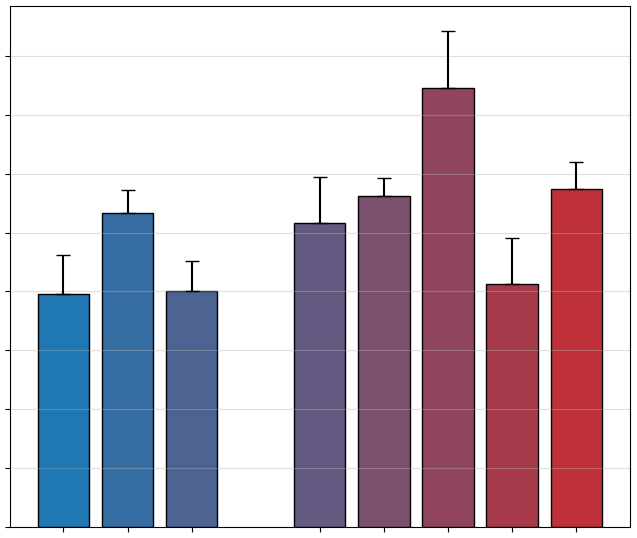


**2.50**

**5.00**

**7.50**

**10.0**

**12.5**

**15.0**

**17.5**

**20.0**

**A)**


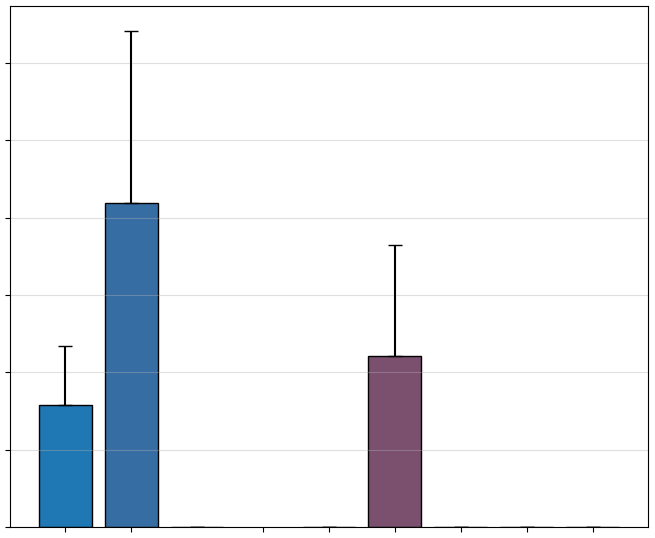


**0.1**

**0.2**

**0.3**

**0.4**

**0.6**

**0.5**

**B)**


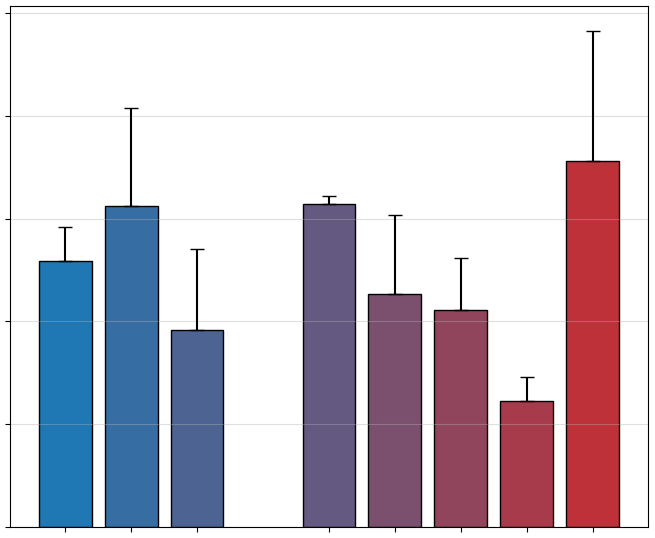


**0.5**

**1.0**

**1.5**

**2.5**

**2.0**


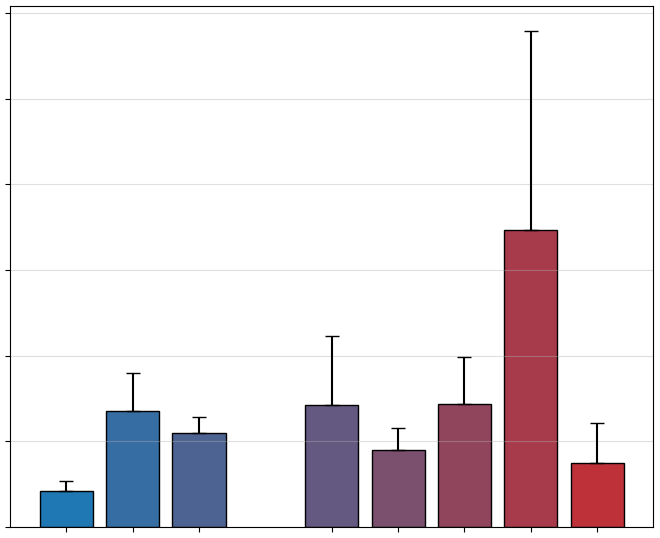


**12**

**10**

**8**

**0**

**6**

**4**

**2**

**#**

**#**

**#**

**C)**

**D)**

Supplementary Figure 6. Changes in the turnover level of 3-MT/DA assessed after intracerebral administration into ST of five increasing doses of Atsttrin using stereotactic methods in C57BL/6 mice subjected to MPTP intoxication within ST (A), CA (B), CX (C) and CM (D). The results were expressed as the value of the concentration ratio of the analyzed monoamines. The results were presented as mean values ± SEM. * - difference from the appropriate control group (K2), *p<0.05, **p<0.01, ***p<0.001; # - difference from the appropriate control group (K3), ^#^p<0.05; ^##^p<0.01; ^###^p<0.001

**K1**

**K2**

**K3**

**P1**

**P2**

**P3**

**P4**

**P5**

**K1**

**K2**

**K3**

**P1**

**P2**

**P3**

**P4**

**P5**

**K1**

**K2**

**K3**

**P1**

**P2**

**P3**

**P4**

**P5**

**K1**

**K2**

**K3**

**P1**

**P2**

**P3**

**P4**

**P5**

**0**

**Control group not subjected to any intervention (7d)**

**4x MPTP-HCL (7d)**

**Ringer’s solution (4 μL) → ST + 4x MPTP-HCL (7d)**

**Atsttrin 0.1 μg/4 μL (0.025 μg/μL) → ST + 4x MPTP-HCL (7d)**

**3-MT/DA turnover – ST**

**0**

**Atsttrin 0.5 μg/4 μL (0.125 μg/μL) → ST + 4x MPTP-HCL (7d)**

**Atsttrin 1 μg/4 μL (0.25 μg/μL) → ST + 4x MPTP-HCL (7d)**

**Atsttrin 5 μg/4 μL (1.25 μg/μL) → ST + 4x MPTP-HCL (7d)**

**x**

**TP**

**(**

**)**

**L (7d)**

**Kontrola niepoddana żadnej procedurze i int**

**rwencji (7d)**

*****

*****

**D)**

**B)**

**A)**

**Atsttrin 2 μg/4 μLl (0.5 μg/μL) → ST + 4x MPTP-HCL (7d)**

**(0.5 μg**

**μ**

**)**

**4**

**P**

**C**

**L (7d)**

**Kontrola niepoddana żadnej procedurze i int**

**rwencji (7d)**

*****

*****

**D)**

**B)**

**A)**

**3-MT/DA turnover – CA**

**3-MT/DA turnover – CX**

**3-MT/DA turnover – CM**

**3-MT/DA ratio**

**3-MT/DA ratio**

**3-MT/DA ratio**

**3-MT/DA ratio**

**0**

**##**

**#**

**#**

**0.05**

**0.10**

**0.15**

**0.20**

**0.25**

**0.30**

**0.35**

**0.40**

**0.6**

**0.8**

**1.0**

**1.2**

**1.6**

**1.4**

**0.5**

**1.0**

**1.5**

**2.5**

**2.0**


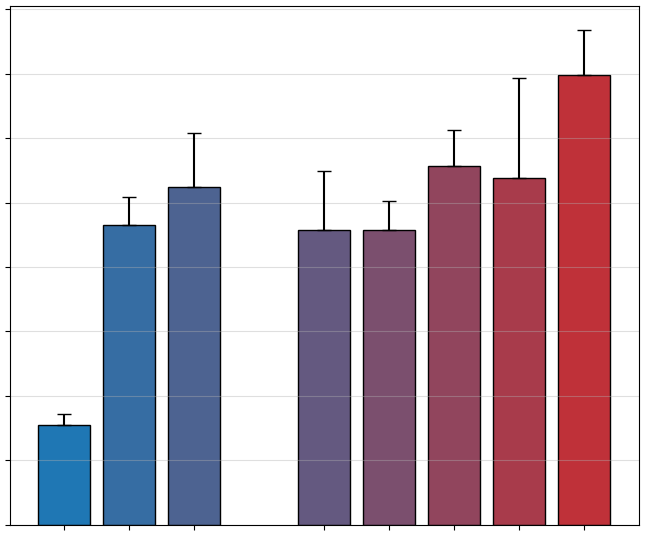

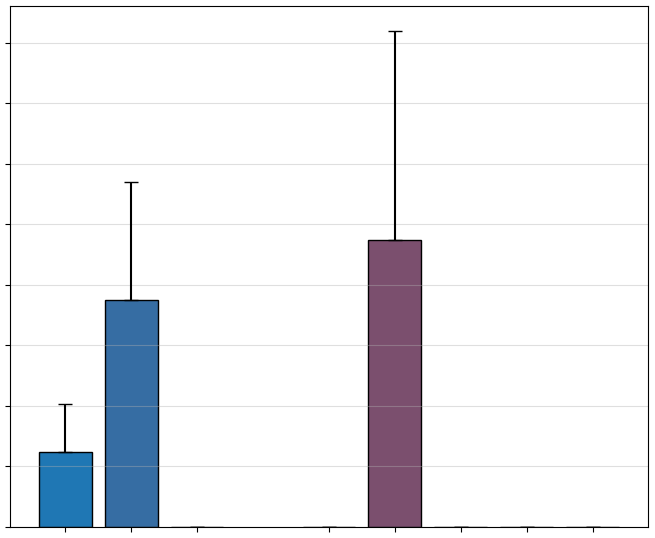


**0.4**

**0.2**

**B)**

**A)**


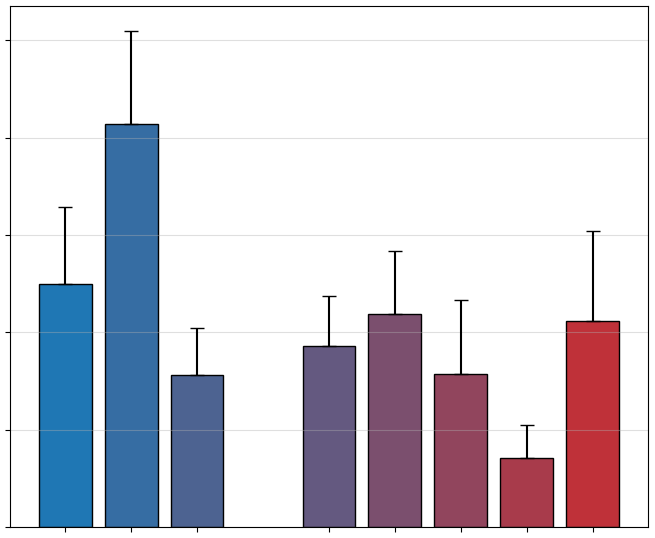


**C)**


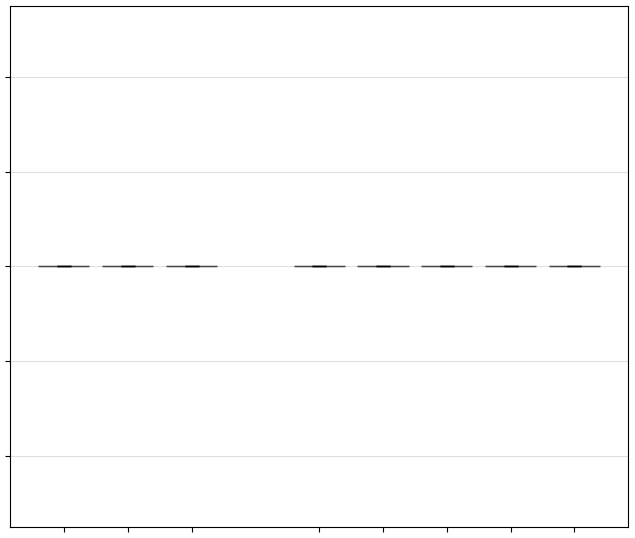


**D)**

**-0.04**

**-0.02**

**0.02**

**0.04**

**0**

*****

******

Supplementary Figure 7. Changes in the turnover level of HVA/DA assessed after intracerebral administration into ST of five increasing doses of Atsttrin using stereotactic methods in C57BL/6 mice subjected to MPTP intoxication within ST (A), CA (B), CX (C) and CM (D). The results were expressed as the value of the concentration ratio of the analyzed monoamines. The results were presented as mean values ± SEM. * - difference from the appropriate control group (K2), *p<0.05, **p<0.01, ***p<0.001; # - difference from the appropriate control group (K3), ^#^p<0.05; ^##^p<0.01; ^###^p<0.001

**K1**

**K2**

**K3**

**P1**

**P2**

**P3**

**P4**

**P5**

**K1**

**K2**

**K3**

**P1**

**P2**

**P3**

**P4**

**P5**

**K1**

**K2**

**K3**

**P1**

**P2**

**P3**

**P4**

**P5**

**K1**

**K2**

**K3**

**P1**

**P2**

**P3**

**P4**

**P5**

**0**

**0**

**Control group not subjected to any intervention (7d)**

**4x MPTP-HCL (7d)**

**Ringer’s solution (4 μL) → ST + 4x MPTP-HCL (7d)**

**Atsttrin 0.1 μg/4 μL (0.025 μg/μL) → ST + 4x MPTP-HCL (7d)**

**HVA/DA turnover – ST**

**0**

**Atsttrin 0.5 μg/4 μL (0.125 μg/μL) → ST + 4x MPTP-HCL (7d)**

**Atsttrin 1 μg/4 μL (0.25 μg/μL) → ST + 4x MPTP-HCL (7d)**

**Atsttrin 5 μg/4 μL (1.25 μg/μL) → ST + 4x MPTP-HCL (7d)**

**x**

**TP**

**(**

**)**

**L (7d)**

**Kontrola niepoddana żadnej procedurze i int**

**rwencji (7d)**

*****

*****

**D)**

**B)**

**A)**

**Atsttrin 2 μg/4 μL (0.5 μg/μL) → ST + 4x MPTP-HCL (7d)**

**(0.5 μg**

**μ**

**)**

**4**

**P**

**C**

**L (7d)**

**Kontrola niepoddana żadnej procedurze i int**

**rwencji (7d)**

*****

*****

**D)**

**B)**

**A)**

**HVA/DA turnover – CA**

**HVA/DA turnover – CX**

**HVA/DA turnover – CM**

**HVA/DA ratio**

**HVA/DA ratio**

**HVA/DA ratio**

**HVA/DA ratio**

**0.3**

**0.5**

*****

**#**

**0.4**


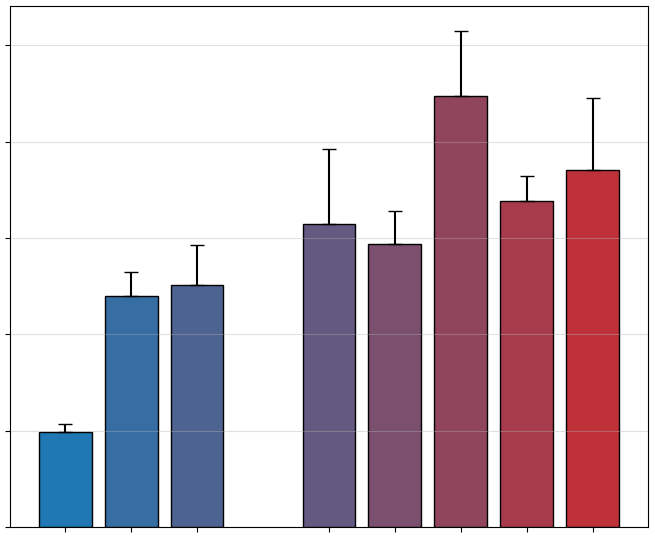


**0.2**

**0.1**


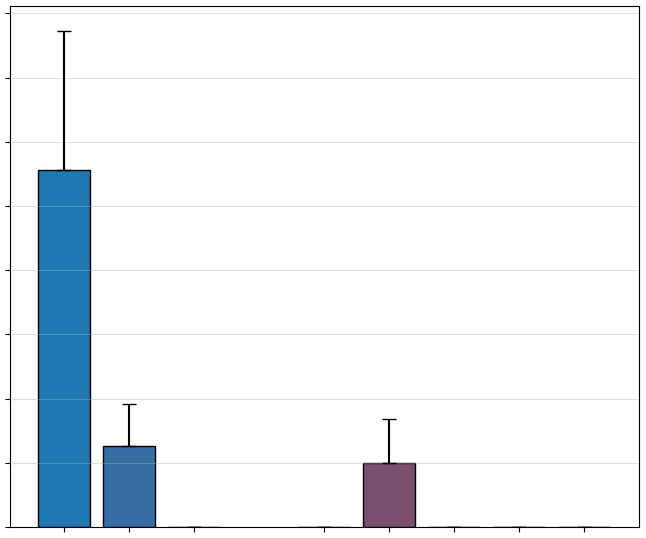


**2.00**

**1.75**

**1.50**

**1.25**

**1.00**

**0.75**

**0.50**

**0.25**


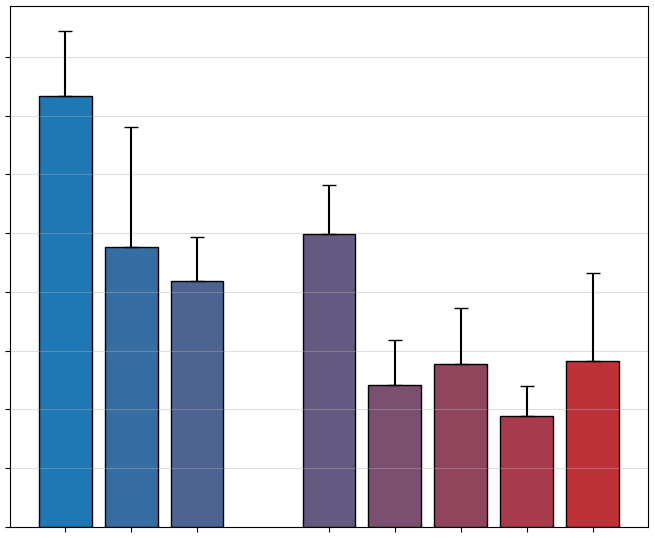


**A)**

**B)**

**C)**

**3.0**

**4.0**

**3.5**

**2.5**

**2.0**

**1.5**

**1.0**

**0.5**


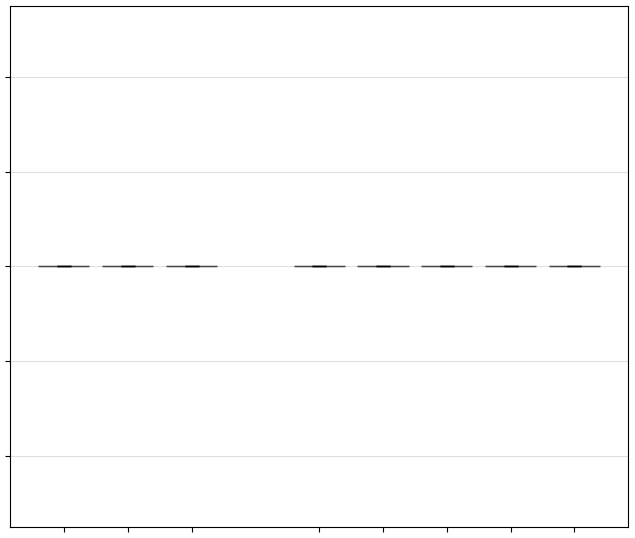


**-0.04**

**-0.02**

**0**

**0.02**

**0.04**

**D)**

*****

**^#^**

*****

**#**

Supplementary Figure 8. Changes in the concentration of NA assessed after intracerebral administration into ST of five increasing doses of Atsttrin using stereotactic methods in C57BL/6 mice subjected to MPTP intoxication within ST (A), CA (B), CX (C) and CM (D). The sample concentration was expressed as pg/mg wet tissue. The results were presented as mean values ± SEM. * - difference from the appropriate control group (K2), *p<0.05, **p<0.01, ***p<0.001; # - difference from the appropriate control group (K3), ^#^p<0.05; ^##^p<0.01; ^###^p<0.001

**K1**

**K2**

**K3**

**P1**

**P2**

**P3**

**P4**

**P5**

**K1**

**K2**

**K3**

**P1**

**P2**

**P3**

**P4**

**P5**

**K1**

**K2**

**K3**

**P1**

**P2**

**P3**

**P4**

**P5**

**K1**

**K2**

**K3**

**P1**

**P2**

**P3**

**P4**

**P5**

**0**

**0**

**0**

**Control group not subjected to any intervention (7d)**

**4x MPTP-HCL (7d)**

**Ringer’s solution (4 μL) → ST + 4x MPTP-HCL (7d)**

**Atsttrin 0.1 μg/4 μL (0.025 μg/μL) → ST + 4x MPTP-HCL (7d)**

**NA concentration – ST**

**0**

**Atsttrin 0.5 μg/4 μL (0.125 μg/μL) → ST + 4x MPTP-HCL (7d)**

**Atsttrin 1 μg/4 μL (0.25 μg/μL) → ST + 4x MPTP-HCL (7d)**

**Atsttrin 5 μg/4 μL (1.25 μg/μL) → ST + 4x MPTP-HCL (7d)**

**x**

**TP**

**(**

**)**

**L (7d)**

**Kontrola niepoddana żadnej procedurze i int**

**rwencji (7d)**

*****

*****

**D)**

**B)**

**A)**

**Atsttrin 2 μg/4 μL (0.5 μg/μL) → ST + 4x MPTP-HCL (7d)**

**(0.5 μg**

**μ**

**)**

**4**

**P**

**C**

**L (7d)**

**Kontrola niepoddana żadnej procedurze i int**

**rwencji (7d)**

*****

*****

**D)**

**B)**

**A)**

**NA concentration – CA**

**NA concentration – CX**

**NA concentration – CM**

**NA concentration [pg/mg]**

**NA concentration [pg/mg]**

**NA concentration [pg/mg]**

**NA concentration [pg/mg]**

**60**

**100**

**80**

**40**

**20**

**500**

**D)**


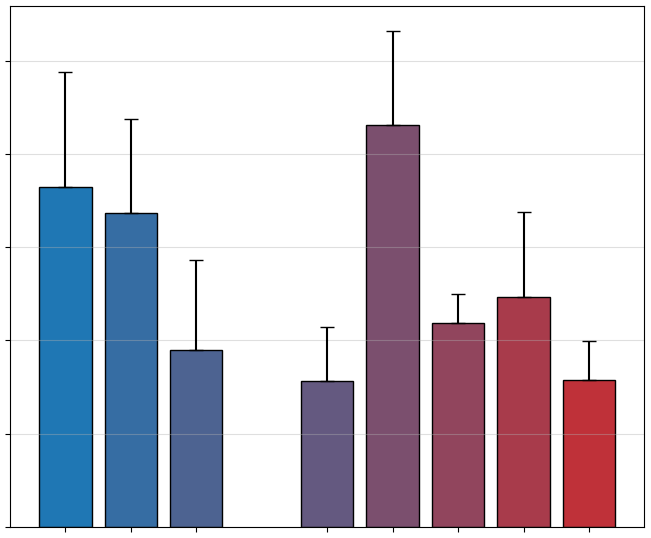


**A)**


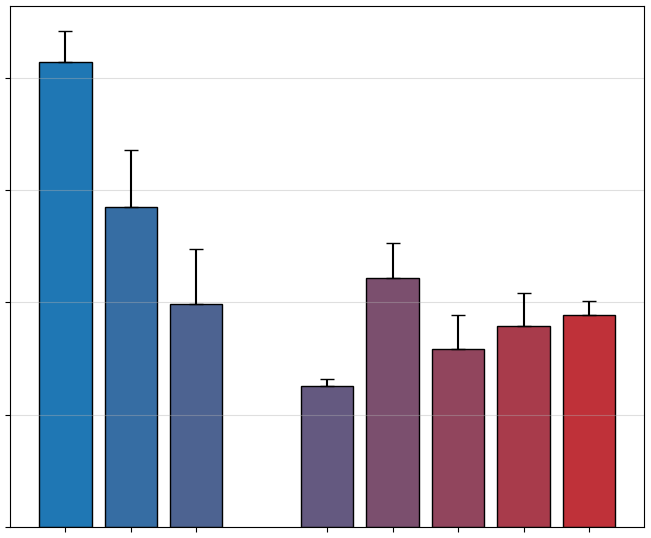


**B)**

**400**

**300**

**200**

**100**


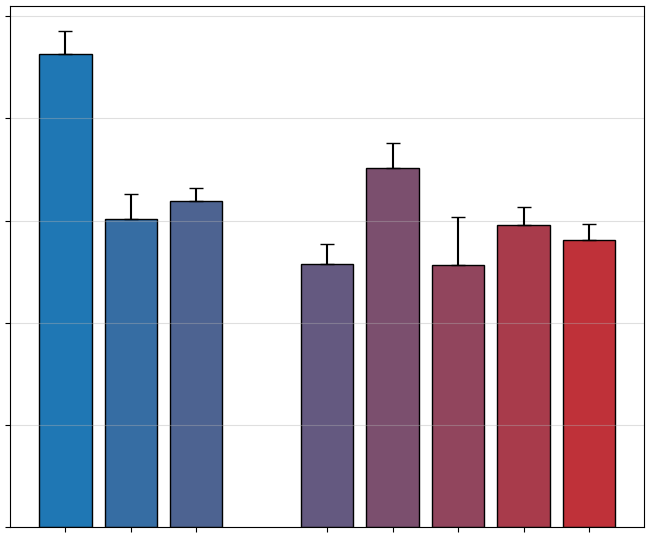


**400**

**300**

**200**

**100**

**C)**


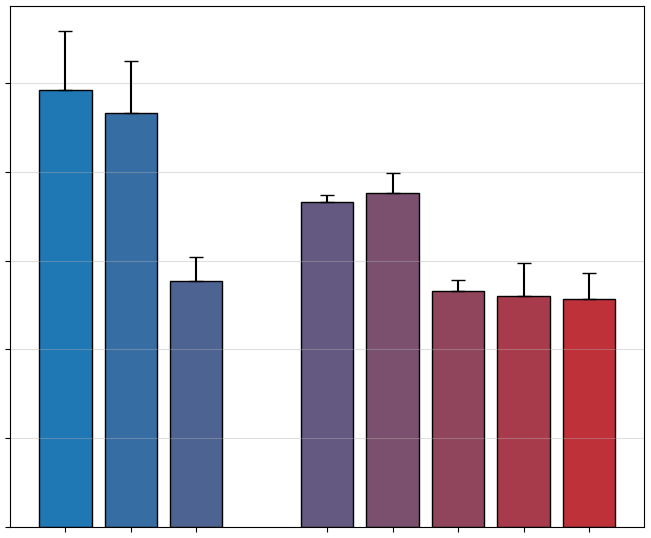


**250**

**200**

**150**

**100**

**50**

*****

**#**

**#**

**#**

*****

*****

******

**K1**

**K2**

**K3**

**P1**

**P2**

**P3**

**P4**

**P5**

**K1**

**K2**

**K3**

**P1**

**P2**

**P3**

**P4**

**P5**

**K1**

**K2**

**K3**

**P1**

**P2**

**P3**

**P4**

**P5**

**K1**

**K2**

**K3**

**P1**

**P2**

**P3**

**P4**

**P5**

**0**

**0**

**0**

**Control group not subjected to any intervention (7d)**

**4x MPTP-HCL (7d)**

**Ringer’s solution (4 μL) → ST + 4x MPTP-HCL (7d)**

**Atsttrin 0.1 μg/4 μL (0.025 μg/μL) → ST + 4x MPTP-HCL (7d)**

**MHPG concentration – ST**

**0**

**Atsttrin 0.5 μg/4 μL (0.125 μg/μL) → ST + 4x MPTP-HCL (7d)**

**Atsttrin 1 μg/4 μL (0.25 μg/μL) → ST + 4x MPTP-HCL (7d)**

**Atsttrin 5 μg/4 μL (1.25 μg/μL) → ST + 4x MPTP-HCL (7d)**

**x**

**TP**

**(**

**)**

**L (7d)**

**Kontrola niepoddana żadnej procedurze i int**

**rwencji (7d)**

*****

*****

**D)**

**B)**

**A)**

**Atsttrin 2 μg/4 μL (0.5 μg/μL) → ST + 4x MPTP-HCL (7d)**

**(0.5 μg**

**μ**

**)**

**4**

**P**

**C**

**L (7d)**

**Kontrola niepoddana żadnej procedurze i int**

**rwencji (7d)**

*****

*****

**D)**

**B)**

**A)**

**MHPG concentration – CA**

**MHPG concentration – CX**

**MHPG concentration – CM**

**MHPG concentration [pg/mg]**

**MHPG concentration [pg/mg]**

**MHPG concentration [pg/mg]**

**MHPG concentration [pg/mg]**

**300**

**50**


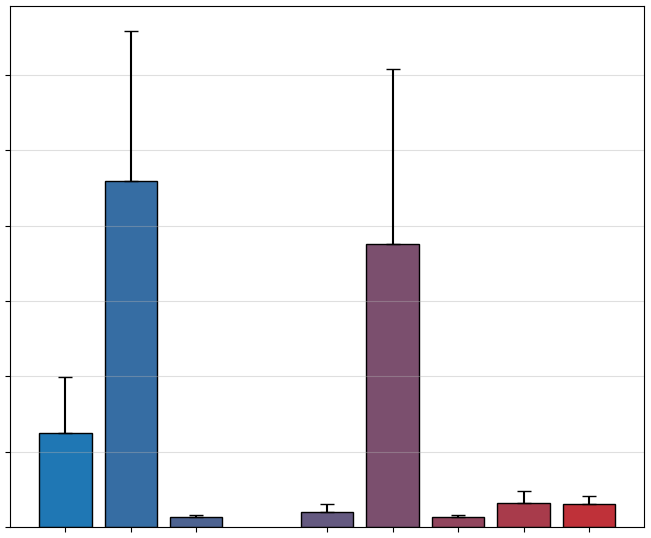


**250**

**200**

**150**

**100**

**A)**


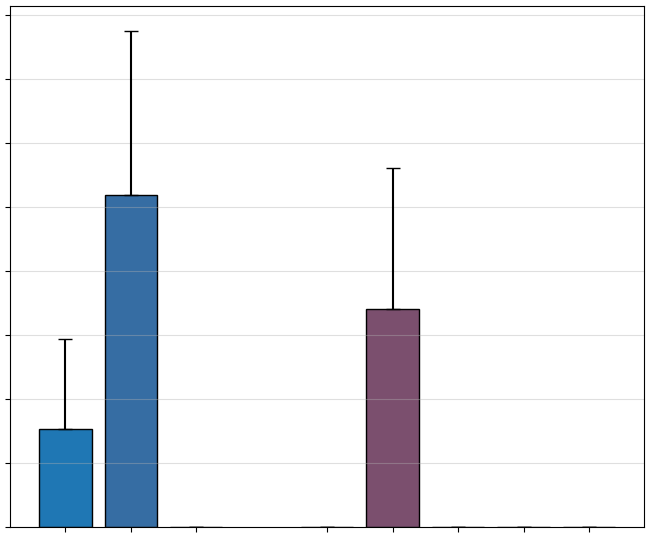


**400**

**350**

**300**

**250**

**200**

**150**

**100**

**50**

**B)**


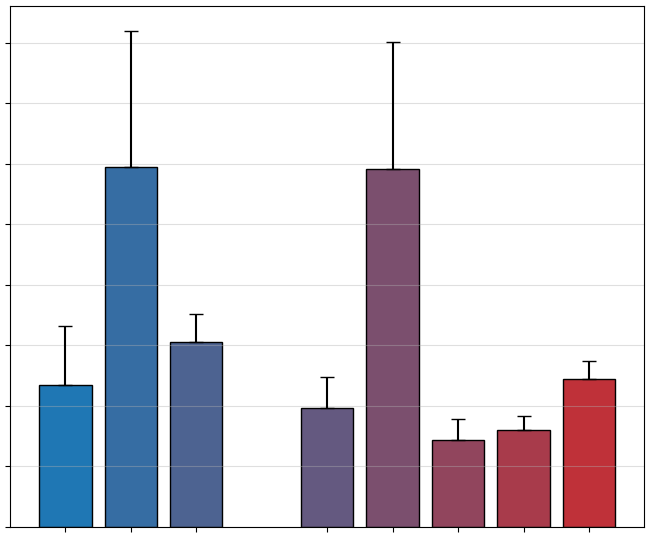


**400**

**350**

**300**

**250**

**200**

**150**

**100**

**50**

**C)**


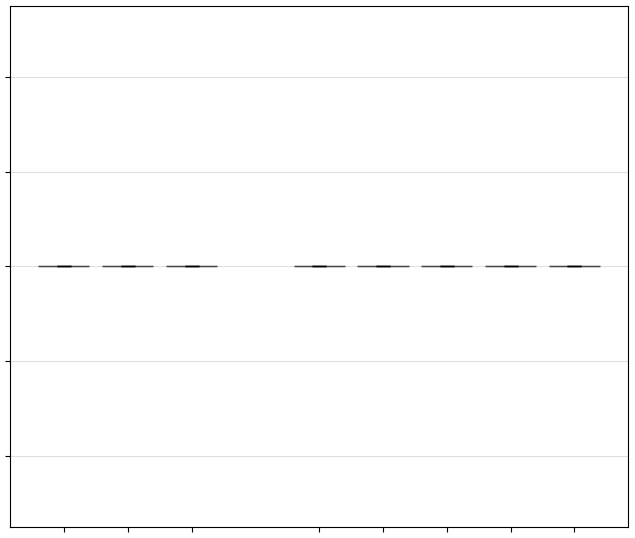


**D)**

**-0.04**

**-0.02**

**0.02**

**0.04**

**0**

**#**

**#**

Supplementary Figure 9. Changes in the concentration of MHPG assessed after intracerebral administration into ST of five increasing doses of Atsttrin using stereotactic methods in C57BL/6 mice subjected to MPTP intoxication within ST (A), CA (B), CX (C) and CM (D). The sample concentration was expressed as pg/mg wet tissue. The results were presented as mean values ± SEM. * - difference from the appropriate control group (K2), *p<0.05, **p<0.01, ***p<0.001; # - difference from the appropriate control group (K3), ^#^p<0.05; ^##^p<0.01; ^###^p<0.001

**K1**

**K2**

**K3**

**P1**

**P2**

**P3**

**P4**

**P5**

**K1**

**K2**

**K3**

**P1**

**P2**

**P3**

**P4**

**P5**

**K1**

**K2**

**K3**

**P1**

**P2**

**P3**

**P4**

**P5**

**K1**

**K2**

**K3**

**P1**

**P2**

**P3**

**P4**

**P5**

**0**

**Control group not subjected to any intervention (7d)**

**4x MPTP-HCL (7d)**

**Ringer’s solution (4 μL) → ST + 4x MPTP-HCL (7d)**

**Atsttrin 0.1 μg/4 μL (0.025 μg/μL) → ST + 4x MPTP-HCL (7d)**

**MHPG/NA turnover – ST**

**0**

**Atsttrin 0.5 μg/4 μL (0.125 μg/μL) → ST + 4x MPTP-HCL (7d)**

**Atsttrin 1 μg/4 μL (0.25 μg/μL) → ST + 4x MPTP-HCL (7d)**

**Atsttrin 5 μg/4 μL (1.25 μg/μL) → ST + 4x MPTP-HCL (7d)**

**x**

**TP**

**(**

**)**

**L (7d)**

**Kontrola niepoddana żadnej procedurze i int**

**rwencji (7d)**

*****

*****

**D)**

**B)**

**A)**

**Atsttrin 2 μg/4 μL (0.5 μg/μL) → ST + 4x MPTP-HCL (7d)**

**(0.5 μg**

**μ**

**)**

**4**

**P**

**C**

**L (7d)**

**Kontrola niepoddana żadnej procedurze i int**

**rwencji (7d)**

*****

*****

**D)**

**B)**

**A)**

**MHPG/NA turnover – CA**

**MHPG/NA turnover – CX**

**MHPG/NA turnover – CM**

**MHPG/NA ratio**

**MHPG/NA ratio**

**MHPG/NA ratio**

**MHPG/NA ratio**

**0**

**##**

**#**

**#**

**1.0**

**B)**

**0.4**

**0.6**

**0.8**

**1.2**

**1.0**

**0**


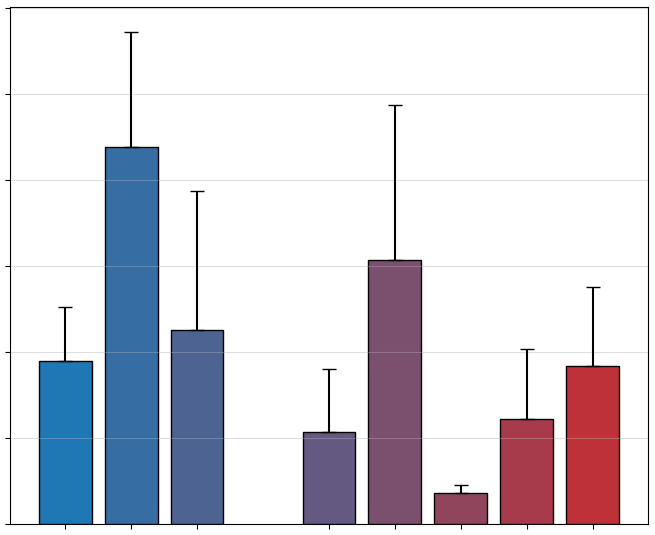


**2.5**

**A)**

**2.0**

**1.5**

**1.0**

**0.5**


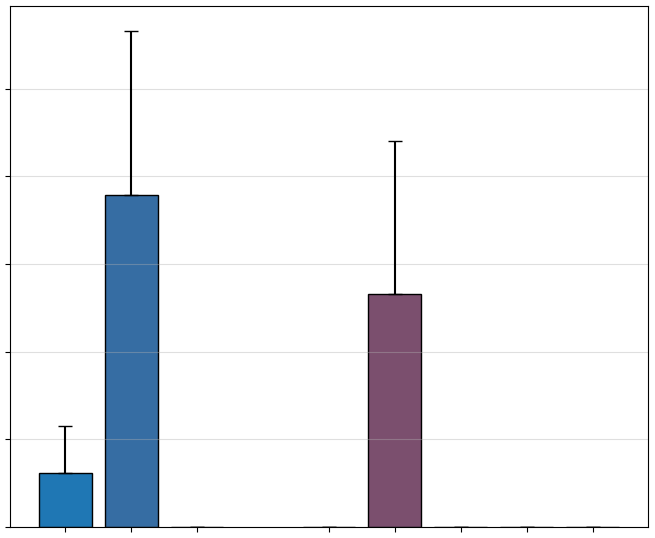


**0.8**

**0.6**

**0.4**

**0.2**


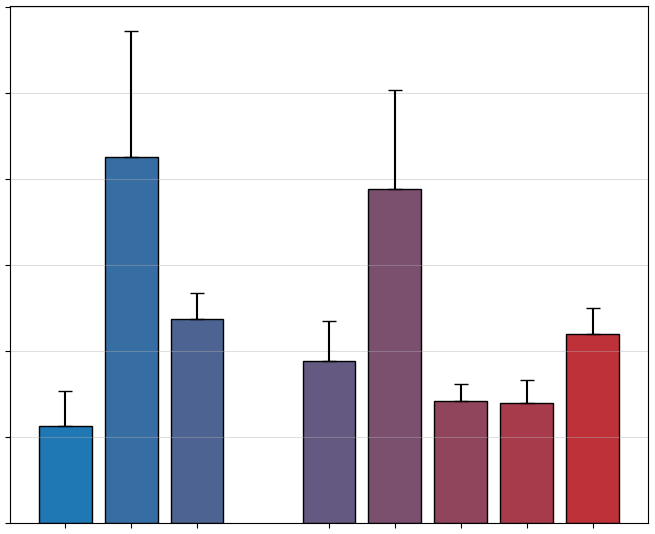


**C)**

**0.2**


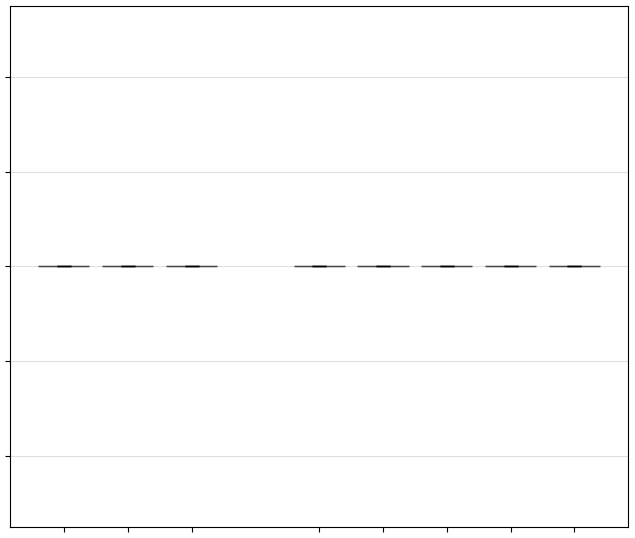


**D)**

**-0.04**

**-0.02**

**0.02**

**0.04**

**0**

*****

**#**

**#**

**3.0**

Supplementary Figure 10. Changes in the turnover level of MHPG/NA assessed after intracerebral administration into ST of five increasing doses of Atsttrin using stereotactic methods in C57BL/6 mice subjected to MPTP intoxication within ST (A), CA (B), CX (C) and CM (D). The results were expressed as the value of the concentration ratio of the analyzed monoamines. The results were presented as mean values ± SEM. * - difference from the appropriate control group (K2), *p<0.05, **p<0.01, ***p<0.001; # - difference from the appropriate control group (K3), ^#^p<0.05; ^##^p<0.01; ^###^p<0.001

**K1**

**K2**

**K3**

**P1**

**P2**

**P3**

**P4**

**P5**

**K1**

**K2**

**K3**

**P1**

**P2**

**P3**

**P4**

**P5**

**K1**

**K2**

**K3**

**P1**

**P2**

**P3**

**P4**

**P5**

**K1**

**K2**

**K3**

**P1**

**P2**

**P3**

**P4**

**P5**

**0**

**0**

**0**

*****

**Control group not subjected to any intervention (7d)**

**4x MPTP-HCL (7d)**

**Ringer’s solution (4 μL) → ST + 4x MPTP-HCL (7d)**

**Atsttrin 0.1 μg/4 μL (0.025 μg/μL) → ST + 4x MPTP-HCL (7d)**

**5-HT concentration – ST**

**0**

**Atsttrin 0.5 μg/4 μL (0.125 μg/μL) → ST + 4x MPTP-HCL (7d)**

**Atsttrin 1 μg/4 μL (0.25 μg/μL) → ST + 4x MPTP-HCL (7d)**

**Atsttrin 5 μg/4 μL (1.25 μg/μL) → ST + 4x MPTP-HCL (7d)**

**x**

**TP**

**(**

**)**

**L (7d)**

**Kontrola niepoddana żadnej procedurze i int**

**rwencji (7d)**

*****

*****

**D)**

**B)**

**A)**

**Atsttrin 2 μg/4 μL (0.5 μg/μL) → ST + 4x MPTP-HCL (7d)**

**(0.5 μg**

**μ**

**)**

**4**

**P**

**C**

**L (7d)**

**Kontrola niepoddana żadnej procedurze i int**

**rwencji (7d)**

*****

*****

**D)**

**B)**

**A)**

**5-HT concentration – CA**

**5-HT concentration – CX**

**5-HT concentration – CM**

**5-HT concentration [pg/mg]**

**5-HT concentration [pg/mg]**

**5-HT concentration [pg/mg]**

**5-HT concentration [pg/mg]**

**600**

*****

*****

**#**

**500**


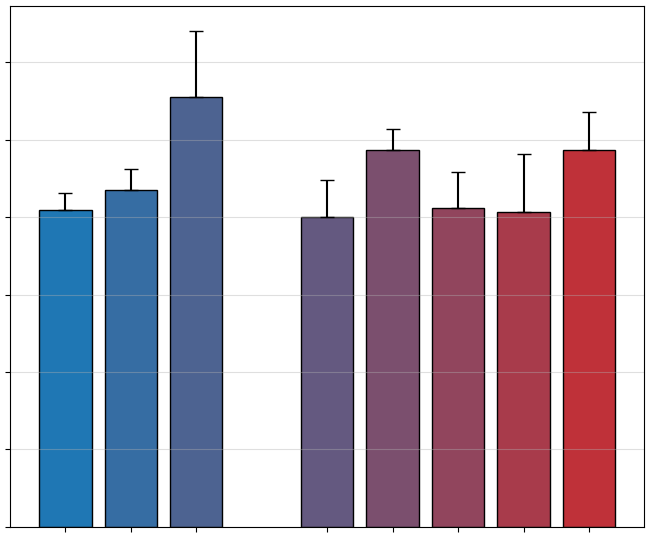


**400**

**300**

**200**

**100**

**A)**


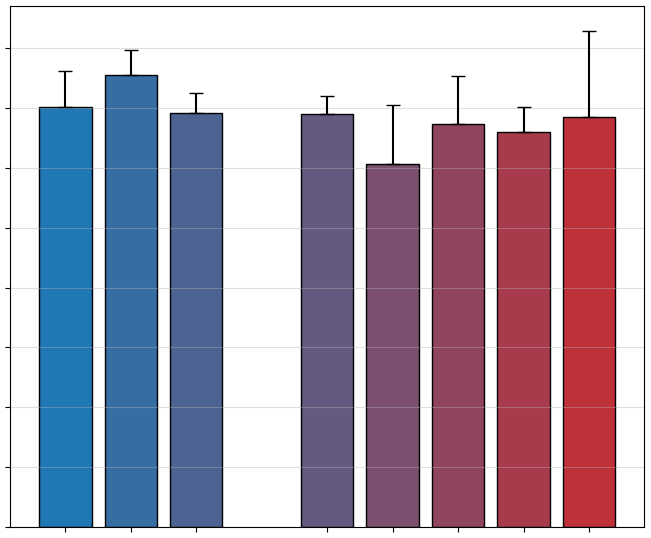


**800**

**700**

**600**

**500**

**400**

**300**

**200**

**100**

**B)**


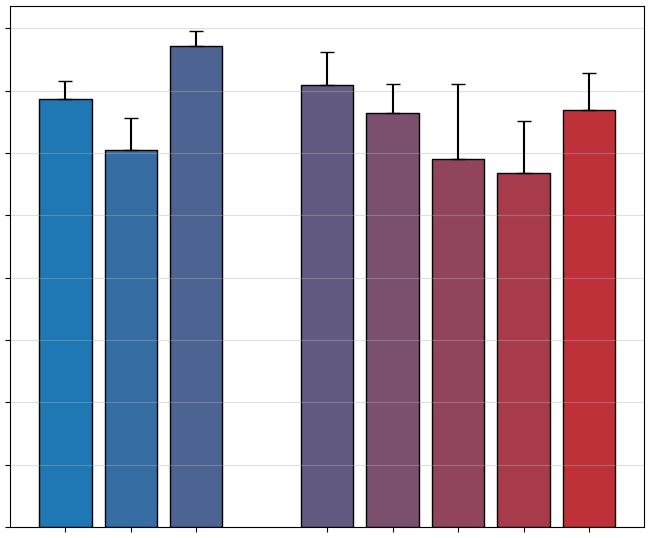


**800**

**700**

**600**

**500**

**400**

**300**

**200**

**100**

**C)**


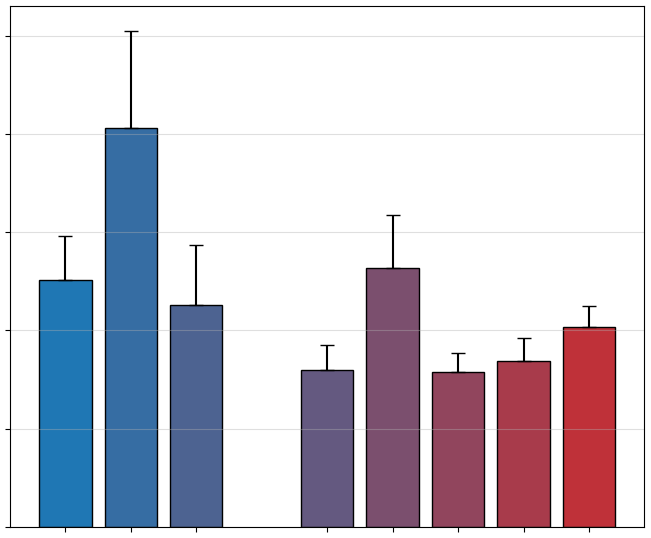


**500**

**400**

**300**

**200**

**100**

*****

*****

*****

**D)**

Supplementary Figure 11. Changes in the concentration of 5-HT assessed after intracerebral administration into ST of five increasing doses of Atsttrin using stereotactic methods in C57BL/6 mice subjected to MPTP intoxication within ST (A), CA (B), CX (C) and CM (D). The sample concentration was expressed as pg/mg wet tissue. The results were presented as mean values ± SEM. * - difference from the appropriate control group (K2), *p<0.05, **p<0.01, ***p<0.001; # - difference from the appropriate control group (K3), ^#^p<0.05; ^##^p<0.01; ^###^p<0.001

**K1**

**K2**

**K3**

**P1**

**P2**

**P3**

**P4**

**P5**

**K1**

**K2**

**K3**

**P1**

**P2**

**P3**

**P4**

**P5**

**K1**

**K2**

**K3**

**P1**

**P2**

**P3**

**P4**

**P5**

**K1**

**K2**

**K3**

**P1**

**P2**

**P3**

**P4**

**P5**

**0**

**0**

**0**

*****

**Control group not subjected to any intervention (7d)**

**4x MPTP-HCL (7d)**

**Ringer’s solution (4 μL) → ST + 4x MPTP-HCL (7d)**

**Atsttrin 0.1 μg/4 μL (0.025 μg/μL) → ST + 4x MPTP-HCL (7d)**

**5-HIAA concentration – ST**

**0**

**Atsttrin 0.5 μg/4 μL (0.125 μg/μL) → ST + 4x MPTP-HCL (7d)**

**Atsttrin 1 μg/4 μL (0.25 μg/μL) → ST + 4x MPTP-HCL (7d)**

**Atsttrin 5 μg/4 μL (1.25 μg/μL) → ST + 4x MPTP-HCL (7d)**

**x**

**TP**

**(**

**)**

**L (7d)**

**Kontrola niepoddana żadnej procedurze i int**

**rwencji (7d)**

*****

*****

**D)**

**B)**

**A)**

**Atsttrin 2 μg/4 μL (0.5 μg/μL) → ST + 4x MPTP-HCL (7d)**

**(0.5 μg**

**μ**

**)**

**4**

**P**

**C**

**L (7d)**

**Kontrola niepoddana żadnej procedurze i int**

**rwencji (7d)**

*****

*****

**D)**

**B)**

**A)**

**5-HIAA concentration – CA**

**5-HIAA concentration – CX**

**5-HIAA concentration – CM**

**5-HIAA concentration [pg/mg]**

**5-HIAA concentration [pg/mg]**

**5-HIAA concentration [pg/mg]**

**5-HIAA concentration [pg/mg]**

*****

*****

**#**


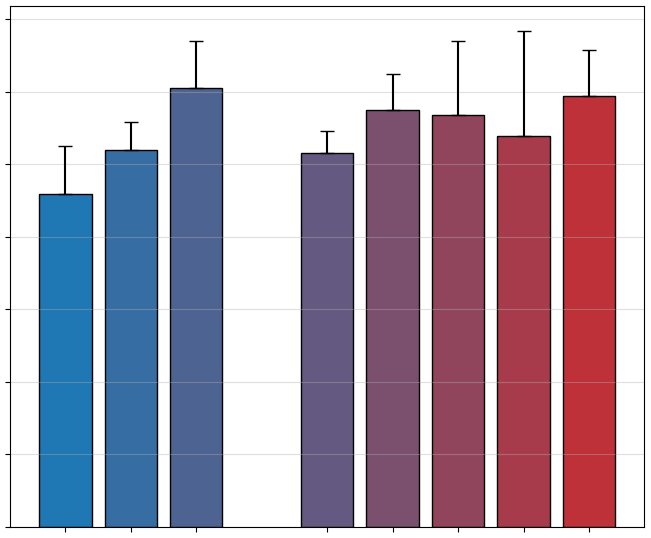


**350**

**300**

**250**

**200**

**150**

**A)**

**100**

**50**


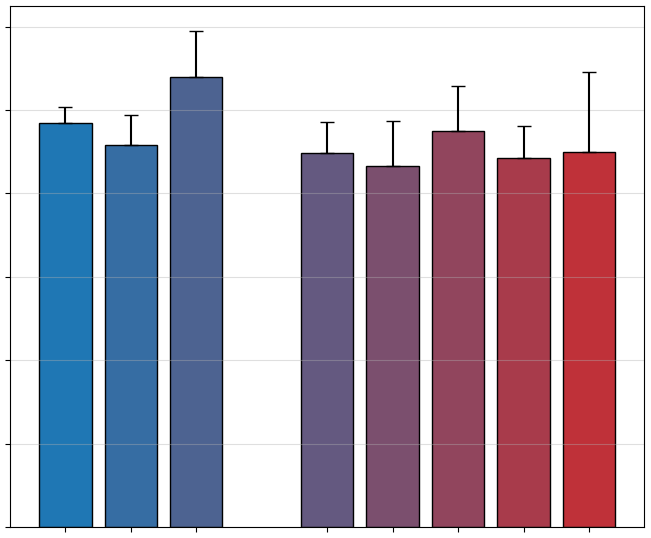


**B)**

**600**

**500**

**400**

**300**

**200**

**100**


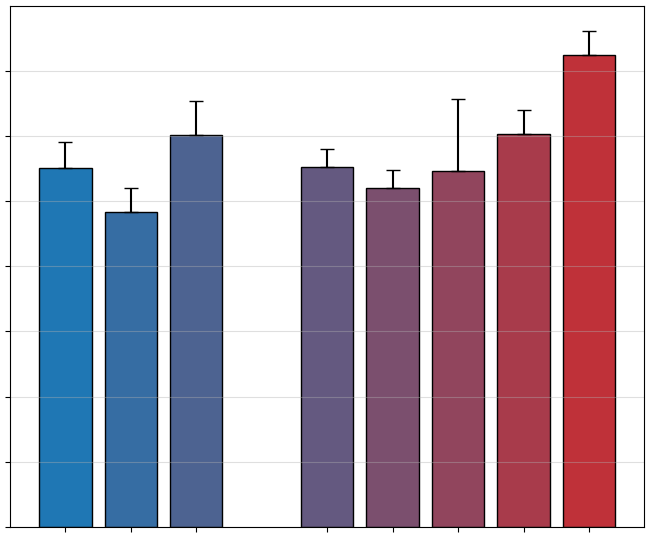


**C)**

**350**

**300**

**250**

**200**

**150**

**100**

**50**


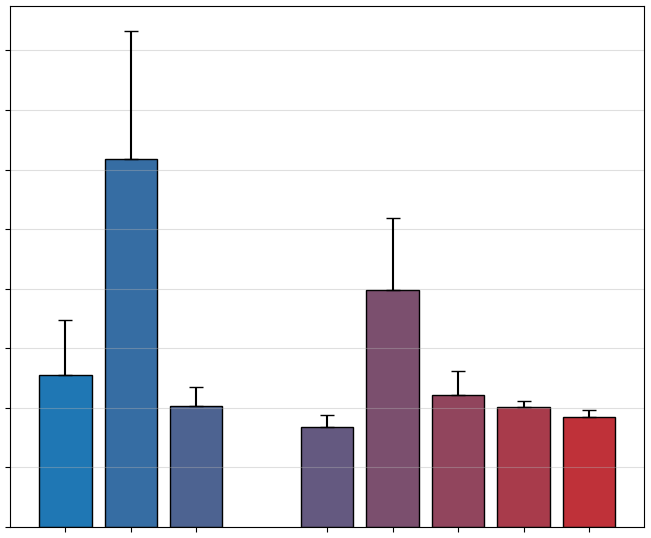


**400**

**350**

**300**

**250**

**200**

**150**

**100**

**50**

*****

*******

**D)**

Supplementary Figure 12. Changes in the concentration of 5-HIAA assessed after intracerebral administration into ST of five increasing doses of Atsttrin using stereotactic methods in C57BL/6 mice subjected to MPTP intoxication within ST (A), CA (B), CX (C) and CM (D). The sample concentration was expressed as pg/mg wet tissue. The results were presented as mean values ± SEM. * - difference from the appropriate control group (K2), *p<0.05, **p<0.01, ***p<0.001; # - difference from the appropriate control group (K3), ^#^p<0.05; ^##^p<0.01; ^###^p<0.001

**K1**

**K2**

**K3**

**P1**

**P2**

**P3**

**P4**

**P5**

**K1**

**K2**

**K3**

**P1**

**P2**

**P3**

**P4**

**P5**

**K1**

**K2**

**K3**

**P1**

**P2**

**P3**

**P4**

**P5**

**K1**

**K2**

**K3**

**P1**

**P2**

**P3**

**P4**

**P5**

**0**

**Control group not subjected to any intervention (7d)**

**4x MPTP-HCL (7d)**

**Ringer’s solution (4 μL) → ST + 4x MPTP-HCL (7d)**

**Atsttrin 0.1 μg/4 μL (0.025 μg/μL) → ST + 4x MPTP-HCL (7d)**

**5-HIAA/5-HT turnover – ST**

**0**

**Atsttrin 0.5 μg/4 μL (0.125 μg/μL) → ST + 4x MPTP-HCL (7d)**

**Atsttrin 1 μg/4 μL (0.25 μg/μL) → ST + 4x MPTP-HCL (7d)**

**Atsttrin 5 μg/4 μL (1.25 μg/μL) → ST + 4x MPTP-HCL (7d)**

**x**

**TP**

**(**

**)**

**L (7d)**

**Kontrola niepoddana żadnej procedurze i int**

**rwencji (7d)**

*****

*****

**D)**

**B)**

**A)**

**Atsttrin 2 μg/4 μL (0.5 μg/μL) → ST + 4x MPTP-HCL (7d)**

**(0.5 μg**

**μ**

**)**

**4**

**P**

**C**

**L (7d)**

**Kontrola niepoddana żadnej procedurze i int**

**rwencji (7d)**

*****

*****

**D)**

**B)**

**A)**

**5-HIAA/5-HT turnover – CA**

**5-HIAA/5-HT turnover – CX**

**5-HIAA/5-HT turnover – CM**

**5-HIAA/5-HT ratio**

**5-HIAA/5-HT ratio**

**5-HIAA/5-HT ratio**

**5-HIAA/5HT ratio**

**0**

**##**

**#**

**#**

**0.2**

**0.4**

**0.6**

**1.0**

**0.8**

**0**


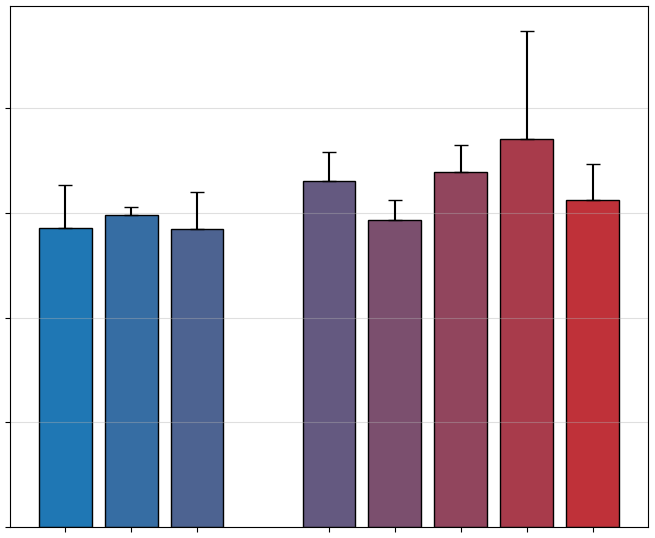


**0.8**

**0.6**

**0.4**

**0.2**


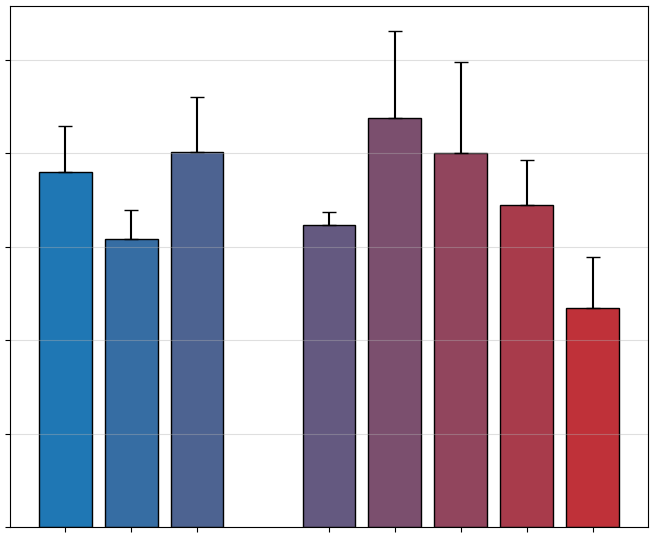


**A)**

**B)**


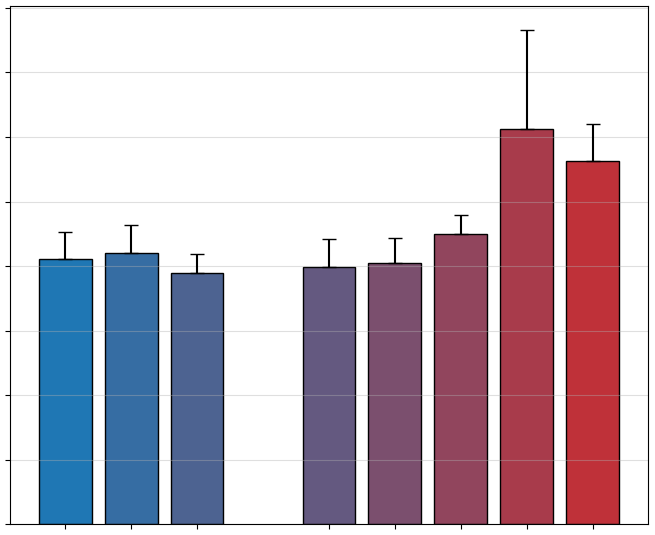


**0.4**

**0.5**

**0.6**

**0.8**

**0.7**

**0.1**

**0.3**

**0.2**

**C)**


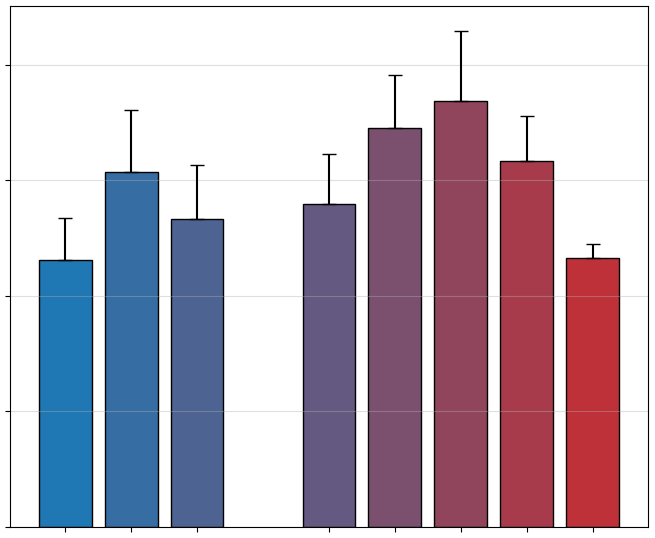


**0.4**

**0.6**

**0.8**

**0.2**

**#**

Supplementary Figure 13. Changes in the turnover level of 5-HIAA/5-HT assessed after intracerebral administration into ST of five increasing doses of Atsttrin using stereotactic methods in C57BL/6 mice subjected to MPTP intoxication within ST (A), CA (B), CX (C) and CM (D). The results were expressed as the value of the concentration ratio of the analyzed monoamines. The results were presented as mean values ± SEM. * - difference from the appropriate control group (K2), *p<0.05, **p<0.01, ***p<0.001; # - difference from the appropriate control group (K3), ^#^p<0.05; ^##^p<0.01; ^###^p<0.001
